# The *Arabidopsis* SWI2/SNF2 Chromatin Remodeler BRAHMA Regulates Polycomb Function during Vegetative Development and Directly Activates the Flowering Repressor Gene *SVP*

**DOI:** 10.1371/journal.pgen.1004944

**Published:** 2015-01-23

**Authors:** Chenlong Li, Chen Chen, Lei Gao, Songguang Yang, Vi Nguyen, Xuejiang Shi, Katherine Siminovitch, Susanne E. Kohalmi, Shangzhi Huang, Keqiang Wu, Xuemei Chen, Yuhai Cui

**Affiliations:** 1 Agriculture and Agri-Food Canada, Southern Crop Protection and Food Research Centre, London, Ontario, Canada; 2 Department of Biology, Western University, London, Ontario, Canada; 3 Department of Botany and Plant Sciences, Institute of Integrative Genome Biology, University of California Riverside, Riverside, California, United States of America; 4 Key Laboratory of Plant Resources Conservation and Sustainable Utilization, South ChinaBotanical Garden, Chinese Academy of Sciences, Guangzhou, Guangdong, China; 5 Clinical Genomics Centre, The UHN/MSH Gene Profiling Facility Mount Sinai Hospital, Toronto, Ontario, Canada; 6 School of Life Sciences, Guangdong Provincial Key Laboratory of Plant Resource, Sun Yat-sen University, Guangzhou, Guangdong, China; 7 Institute of Plant Biology, College of Life Science, National Taiwan University, Taipei, Taiwan; 8 Howard Hughes Medical Institute, University of California Riverside, Riverside, California, United States of America; Heinrich Heine University, UNITED STATES

## Abstract

The chromatin remodeler BRAHMA (BRM) is a Trithorax Group (TrxG) protein that antagonizes the functions of Polycomb Group (PcG) proteins in fly and mammals. Recent studies also implicate such a role for *Arabidopsis* (*Arabidopsis thaliana*) BRM but the molecular mechanisms underlying the antagonism are unclear. To understand the interplay between BRM and PcG during plant development, we performed a genome-wide analysis of trimethylated histone H3 lysine 27 (H3K27me3) in brm mutant seedlings by chromatin immunoprecipitation followed by next generation sequencing (ChIP-seq). Increased H3K27me3 deposition at several hundred genes was observed in brm mutants and this increase was partially supressed by removal of the H3K27 methyltransferase CURLY LEAF (CLF) or SWINGER (SWN). ChIP experiments demonstrated that BRM directly binds to a subset of the genes and prevents the inappropriate association and/or activity of PcG proteins at these loci. Together, these results indicate a crucial role of BRM in restricting the inappropriate activity of PcG during plant development. The key flowering repressor gene *SHORT VEGETATIVE PHASE* (*SVP*) is such a BRM target. In brm mutants, elevated PcG occupancy at *SVP* accompanies a dramatic increase in H3K27me3 levels at this locus and a concomitant reduction of *SVP* expression. Further, our gain- and loss-of-function genetic evidence establishes that BRM controls flowering time by directly activating *SVP* expression. This work reveals a genome-wide functional interplay between BRM and PcG and provides new insights into the impacts of these proteins in plant growth and development.

## Author Summary

In flowering plants, the proper transition from vegetative growth to flowering is critical for their reproductive success and must be controlled precisely. Multiple genes have been shown to regulate the floral transition in response to environmental and endogenous cues. Among them is *SHORT VEGETATIVE PHASE* (*SVP*), a key flowering repressor gene in *Arabidopsis. SVP* is highly expressed during the vegetative phase to promote growth, but the mechanism by which the high expression level of *SVP* is maintained remains unknown. Here, we report a genome-wide study to examine the functional interplay between the BRM chromatin remodeler and the PcG proteins that catalyze trimethylation of lysine 27 on histone H3 (H3K27me3), a histone mark normally associated with transcriptionally repressed genes. We identify BRM as a direct upstream activator of *SVP*. BRM acts to keep the levels of H3K27me3 low at the *SVP* locus by inhibiting the binding and activities of the PcG proteins. Thus, our work identifies a previously unknown mechanism in regulation of flowering time and demonstrates the power of genome-wide approaches in dissecting regulatory networks controlling plant development.

## Introduction

Plant development takes place in distinct phases, each of which is characterized by the activation of a particular set of genes and the repression of others. Precise control of gene expression in each phase is crucial for proper growth and development. The transition from the vegetative to the reproductive phase is controlled precisely by multiple genetic pathways in response to environmental and endogenous signals [1–4]. In *Arabidopsis*, a repressor complex that consists of two MADS box transcription factors, FLOWERING LOCUS C (FLC) and SVP, serves as a negative regulator of flowering time by directly repressing the expression of the floral pathway integrators *FLOWERING LOCUS T* (*FT*) and *SUPPRESSOR OF OVEREXPRESSION OF CO 1* (*SOC1*) [1,5,6]. *SVP* is highly expressed during the vegetative phase [5,7], but is down-regulated during the floral transition by the autonomous and gibberellin (GA) pathways [5], which results in the de-repression of *FT* and *SOC1* to promote flowering. Despite its key role in controlling flowering time, the mechanisms by which *SVP* expression is regulated are still unknown. Particularly, no positive regulator(s) of *SVP* expression in the vegetative phase have been identified.

Polycomb Group (PcG) proteins are epigenetic repressors that maintain the repressed state of genes in cells where the genes should be inactive [8–11]. PcG proteins repress genes through combined activities of at least two multi-protein complexes known as Polycomb Repressive Complex 1 (PRC1) and PRC2 [8]. PRC2 is involved in the establishment and maintenance of the repressed chromatin state, by introducing the H3K27me3 mark. Subsequently, PRC1 binds to the H3K27me3 mark and compacts the chromatin, resulting in the repressed state of PcG target genes. In *Arabidopsis*, at least three forms of PRC2 complexes exist and each of them acts at specific developmental phases [12–15]. CLF and SWINGER (SWN) are two putative H3K27 methyltransferases and act redundantly during the vegetative and reproductive stages [16]. Several thousands of *Arabidopsis* genes were reported to carry the H3K27me3 mark in young seedlings [17–19]. A fraction of PcG target genes was found to carry the H3K27me3 mark specifically in either the shoot apical meristem or leaf cells [18], suggesting dynamic regulation of H3K27me3 deposition. Studies have been carried out to address how PcG proteins deposit H3K27me3 to target genes [12,13,20]. It is less known, however, about the mechanisms by which PcG activities are prevented from targeting certain genes to keep these genes on at particular developmental phases.

SWI/SNF-type chromatin-remodeling protein complexes are thought to utilize energy from ATP hydrolysis to mobilize, disrupt or change nucleosomes to create an open chromatin structure for the access of transcriptional factors or other regulators [21,22]. The SWI2/SNF2 ATPase in *Drosophila*, BRM, was initially classified as a Trithorax group (TrxG) protein since it activates the transcription of homeotic genes and thus antagonizes the function of PcG during fly development [23,24]. However, recent studies indicate that it can either activate or repress target gene expression, through increasing or decreasing the accessibility of the target DNA [24–26], yet its role in the regulation of gene expression is not well understood. Although the biochemical activities of plant SWI/SNF complexes have not been examined, progress has been made to identify the plant SWI/SNF complexes through genetic and molecular analysis [27–30]. In *Arabidopsis*, four SWI2/SNF2 ATPases including BRM and SPLAYED (SYD), four SWI3 proteins (SWI3A to SWI3D), two SWI/SNF ASSOCIATED PROTEINS 73 (SWP73A and SWP73B), two ACTIN RELATED PROTEINS (ARP4 and ARP7), and a single SNF5 subunit termed BUSHY (BSH) were predicted subunits of SWI/SNF complexes [27]. Previous *in vitro* protein-protein interaction data [28,31] and a recent effort in protein complex purification followed by peptide sequencing [32] suggest that these proteins could form several SWI/SNF complexes. Subunits of *Arabidopsis* SWI/SNF complex(es) play crucial roles in many aspects of plant development [26,27,33–36]. The SWP73B (also called BAF60) subunit has been shown to participate in the control of flowering time [37]. The SWI3C protein is involved in gibberellin (GA) responses [38]. *brm* mutants show pleiotropic phenotypes, such as reduced plant size [28,39], downward curling of leaves [28,33], mild floral homeotic defects [28,34], hypersensitivity to abscisic acid [26] and early flowering [33,39,40]. Efforts have been made to understand the reason why *brm* mutants show an early flowering phenotype [40], but the precise role of BRM in flowering time control is still not clear.

Although the roles of PcG proteins and BRM during plant development have been investigated individually, how their activities are coordinated is poorly understood. Interestingly, a recent report in *Arabidopsis* showed that loss of BRM activity led to the increased H3K27me3 at two floral homeotic genes [34], suggesting the antagonistic relationship between BRM and PcG. However, the current model is solely based on the characterization of a few identified targets of BRM, and it remains unknown to what extent BRM is required for antagonizing PcG function in plant. Also the precise mechanism by which BRM antagonizes PcG activity during plant development remains unclear. Finally, whether or not plant BRM might work synergistically with PcG proteins is completely unknown. To address these questions, we have performed a genome-wide analysis of H3K27me3 in *brm* mutant seedlings by chromatin immunoprecipitation followed by next generation sequencing (ChIP-seq). We identify several hundred genes that show increased levels of H3K27me3 upon loss of BRM activity, demonstrating the critical role of BRM in preventing genes from H3K27me3-mediated repression in plant cells. We further show that there is inappropriate invasion of PcG proteins. Finally, by taking advantage of our genome-wide data, we uncover a role for BRM in repressing flowering by activating directly the expression of *SVP*, thus providing an explanation for the early flowering phenotype of *brm* mutants. Together, our results demonstrate that BRM is essential for proper H3K27me3 distribution in the genome and thus plant development.

## Results

### Loss of BRM Activity Leads to the Gain of H3K27me3 at Hundreds of Genes

**Figure 1:**
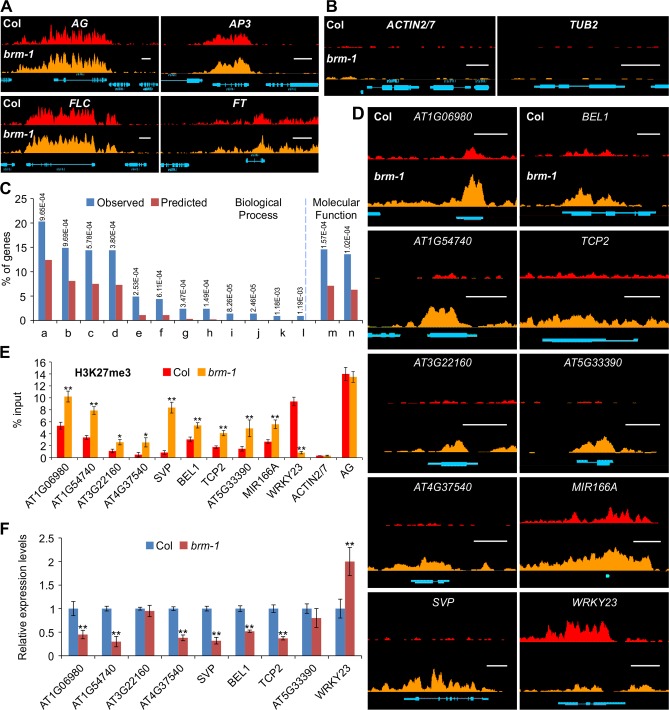
Loss-of-function of *BRM* results in changes of H3K27me3 distribution over several hundred endogenous genes. (A) ChIP-seq data for the well-known H3K27me3 target genes *AG, AP3, FLC* and *FT* from wild type Col (red; top) and *brm-1* (orange; bottom). Gene structures are shown underneath each panel. Scale bars, 1Kb. The plants used were 14-day-old seedlings. (B) ChIP-seq data showing no H3K27me3 signal at two constitutively expressed genes *ACTIN2/7* and *TUB2* in wild-type Col (red; top) and *brm-1* (orange; bottom). Gene structures are shown underneath each panel. Scale bars, 1Kb. (C) Gene Ontology (GO) analysis of the genes showing increased H3K27me3 levels in *brm-1*. Numbers on the top are P values (hypergeometric test) for GO category enrichment generated by comparing the percentage of the corresponding categories in the genes that showed increased H3K27me3 levels with those in the whole genome. (a) Regulation of biological process;(b) Regulation of metabolic process; (c) Regulation of macromolecule metabolic process;(d) Regulation of gene expression; (e) Response to auxin stimulus; (f) Tissue development; (g) Gene silencing by miRNA; (h) Meristem maintenance; (i) Meristem determinacy; (j) Floral meristem determinacy; (k) Leaf shaping; (l) Maintenance of floral meristem identity; (m) Transcription regulator activity; (n) Transcripiton factor activity. (D) ChIP-seq data showing changes in H3K27me3 levels at 10 selected genes in *brm-1*. Nine of them showed an increase and one showed a decrease in H3K27me3 levels. Data for the wild-type Col are shown in red at the top, and *brm-1* is shown in orange at the bottom. Gene structures are shown underneath each panel. Scale bars, 1Kb. (E) ChIP-qPCR validation using independent samples. Data are shown as percentage of input. *ACTIN2/7* and *AG* were used as control loci that exhibited no change in H3K27me3 deposition. Error bars indicate standard deviations from three biological replicates.*: *P* < 0.05; **: *P* < 0.01. (F) Expression analysis of selected genes by qRT-PCR. The expression of each gene was normalized to that of *GAPDH*, and the expression level in Col was set to 1. Error bars indicate standard deviations from three biological replicates.*: *P* < 0.05; **: *P* < 0.01.

To examine whether BRM affects the patterns of H3K27me3 deposition and distribution in a genome-wide scale, we performed ChIP-seq with anti-H3K27me3 antibodies in wild-type Col and *brm-1*, a null allele with a T-DNA insertion [28]. Two independent biological DNA samples were generated and used for sequencing. We mapped the reads to the *Arabidopsis* genome and identified H3K27me3-enriched regions in both wild-type and *brm* mutants. Only H3K27me3-enriched regions identified in both biological replicates were chosen for further data analysis. In 14-day-old wild-type Col seedlings, we identified 5,591 regions, corresponding to 7,230 genes, which were marked by H3K27me3 (S1 Dataset). H3K27me3 target genes identified in our study cover more than 95% (6,322 out of 6634) of those reported in a previous ChIP-seq analysis [19]. Furthermore, in both Col and the *brm-1* mutant, the patterns of H3K27me3 at several well-characterized H3K27me3 target genes, such as *AGAMOUS* (*AG*), *APETALA3* (*AP3*), *FLC* and *FT*, are very similar to those reported by Lu et al [19] (Fig. 1A). In contrast, no H3K27me3 signals at two highly expressed genes, *ACTIN2/7* and *TUB2*, were observed (Fig. 1B), demonstrating the quality and reliability of our ChIP-seq data.

Compared to wild-type, we identified 276 genes at which H3K27me3 levels changed more than twofold in the *brm-1* mutant (see the Materials and Methods section for details). Out of the 276 genes, 258 (93.5%) genes showed more than a twofold increase in H3K27me3 in *brm-1*, while only 18 (6.5%) genes showed more than a twofold reduction in H3K27me3 in *brm-1* (S2 Dataset). Our genome-wide data show that BRM mainly acts to antagonize PRC2 activity during vegetative development, which is consistent with its expected role as a TrxG protein. However, the decreased H3K27me3 at a smaller set of genes in *brm* mutant suggests that BRM could also promote PcG activity at certain loci.

We performed a Gene Ontology (GO) analysis for the genes showing increased H3K27me3 deposition using the BINGO software [41]. In the classification of biological processes, these genes were highly enriched in “regulation of metabolic process” (P = 9.69E-4) and “regulation of gene expression” (P = 3.8E-4; Fig. 1C), and in terms of molecular function, the most enriched category observed was “transcription regulator activity” (P = 1.57E-4). Thus, BRM is involved in a wide spectrum of cellular processes such as gene expression regulation and metabolism through preventing PcG proteins from H3K27me3 deposition. To validate our ChIP-seq data, we randomly chose a subset of genes and performed ChIP followed by quantitative PCR (ChIP-qPCR) using independent chromatin samples. We confirmed the changes in H3K27me3 levels at all 10 selected genes in *brm-1* (Fig. 1D and 1E). We did not detect any marked changes at the PcG non-targets *ACTIN2/7* or the PcG target *AG* (Fig. 1E).

Next, we asked whether the elevated H3K27me3 levels in the *brm-1* mutant caused down-regulation of the corresponding genes. We measured the expression levels of several selected genes that showed increased H3K27me3 levels in *brm-1* by quantitative Reverse Transcription-PCR (qRT-PCR) and observed decreased expression for most but not all of them in *brm-1* (Fig. 1F). Interestingly, we also found increased expression of *WRKY23* (Fig. 1F), a gene with decreased H3K27me3 levels in *brm-1* (Fig. 1D and 1E). These data indicate that there is a positive correlation between increased H3K27me3 levels and decreased gene expression in *brm-1* and also suggest that increased H3K27me3 deposition alone in *brm* might not be sufficient for gene repression at some target loci.

### Removal of CLF or SWN Activity in *brm* Background Results in a Substantial Decrease of H3K27me3 Deposition at Some Genes

**Figure 2:**
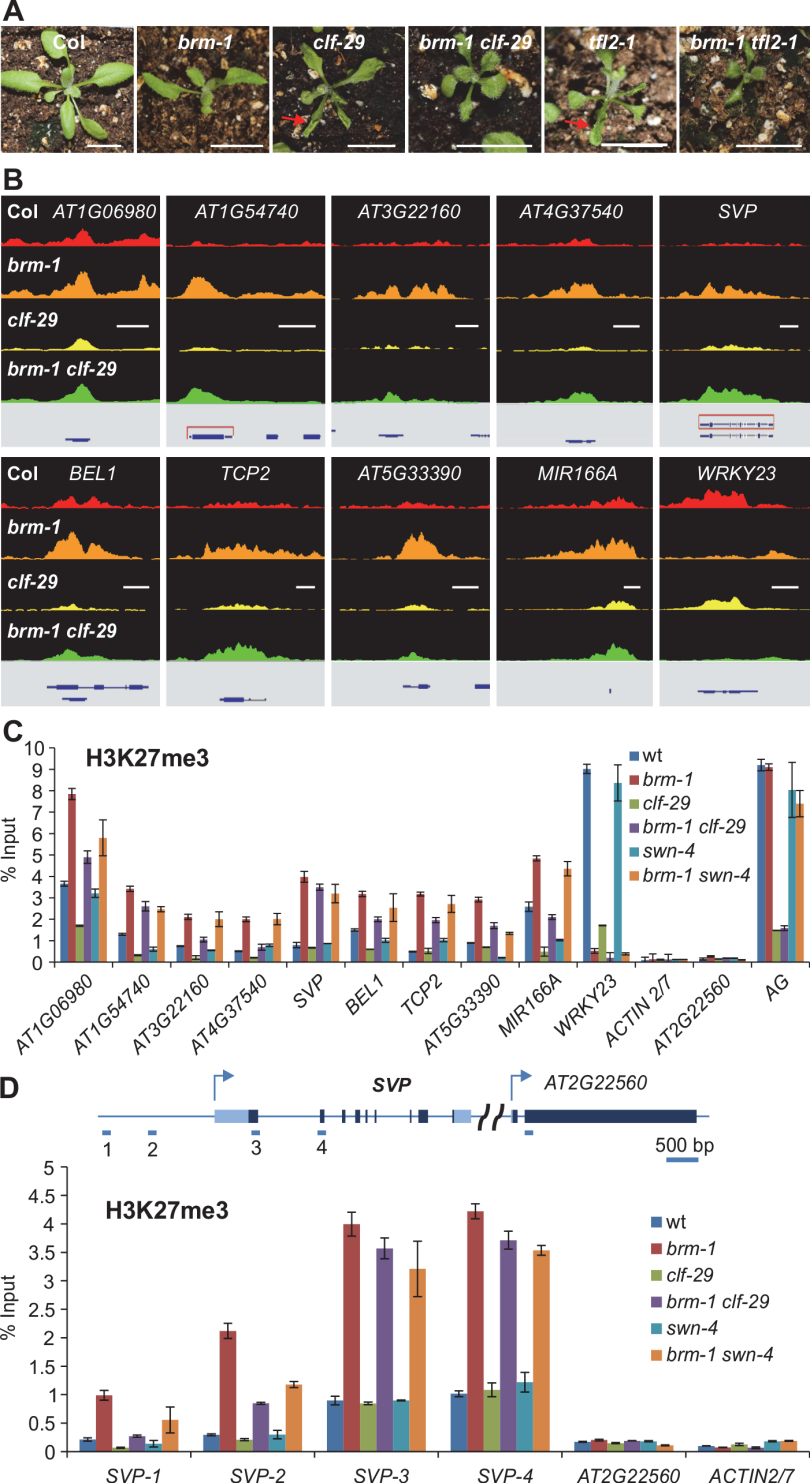
Removal of CLF or SWN activity in *brm* background results in a substantial decrease of H3K27me3 deposition. (A) Loss of BRM activity partially rescues the up-wardly leaf curling phenotypes of *clf-29* and *tfl2-1*. Scale bar: 1 cm. (B) ChIP-seq data comparing H3K27me3 levels at 10 selected genes in Col, *brm-1, clf-29* and *brm-1 clf-29*. Data for the wild type Col are shown in red, *brm-1* in orange, *clf-29* in yellow, and *brm-1 clf-29* in green. Gene structures are shown underneath each panel. Scale bars, 1Kb. (C) ChIP-qPCR validation of the H3K27me3 ChIP-seq data using independent samples; and ChIP-qPCR detection of H3K27me3 levels in *swn-4* and *brm-1 swn-4* mutants. ChIP signals are shown as percentage of input. *ACTIN2/7* and *AT2G22560* (a flanking gene of *SVP*) were used as negative control loci; and *AG* was used as a positive control locus. Error bars indicate standard deviations from three biological replicates. (D) Top panel: schematic representation of the genomic region covering *SVP* and the flanking gene *AT2G22560*. Dark and light blue boxes indicate exon and intron, respectively. Arrows indicate the transcription start sites. Short blue lines indicate the positions of primer pairs used. Bottom panel: ChIP-qPCR determining the levels of H3K27me3 across the *SVP* locus. ChIP signals are shown as percentage of input. Error bars indicate standard deviations from three biological replicates.

In *Arabidopsis*, CLF is thought to be a major H3K27 methyltransferase responsible for the deposition of H3K27me3 in tissues other than seeds [42,43]. LIKE HETEROCHROMATIN PROTEIN 1/TERMINAL FLOWER 2 (LHP1/TFL2) associates with regions with H3K27me3 across the *Arabidopsis* genome and was proposed to be a key component of a plant PRC1 complex [44,45]. Both *clf* and *tfl2* single mutants showed up-ward curling of leaves (Fig. 2A) [42,46]. We reasoned that CLF might be required for the increased H3K27me3 levels at some genes in the *brm-1* mutant. To test this, we first generated a *brm clf* double mutant to examine the genetic relationship between *CLF* and *BRM. clf* single mutants display up-wardly curled leaves while *brm* single mutants show down-ward curling of leaves [28]. Up-ward leaf curling in *clf* mutants can be caused by ectopic expression of floral homeotic genes such as *AG, AP1*, and *AP3* [16,42]. In the *brm clf* double mutants, the up-ward curling of leaves was weaker than that in *clf* single mutants (Fig. 2A and S1 Fig.), suggesting that *brm* can partially suppresses *clf*. We also generated *brm tfl2* double mutants. The leaves of the *brm tfl2* double mutants showed down-ward curling as those in *brm* single mutants (Fig. 2A and S1 Fig.) suggesting that *brm* suppresses *tfl2*’s phenotype of up-wardly curled leaves. These genetic data support a notion that BRM antagonizes PcG function during vegetative development. Consistent with the partially rescued up-ward leaf curling in *brm clf* double mutants, we found decreased ectopic expression of several floral homeotic genes such as *AG, AP1*, and *AP3* in *brm clf* double mutant leaves compared to *clf* single mutants (S2 Fig.). Interestingly, the *brm clf* double mutants were also smaller in terms of overall size than either single mutant, suggesting the additive effect of the two mutations in plant development. Supporting this observation, we noticed that there were more genes mis-regulated in *brm clf* double mutants than either single mutant (S3 Fig.).

To test if CLF is required for the increased H3K27me3 levels at some genes in the *brm-1* mutant, we measured genome-wide H3K27me3 levels in *brm clf* double mutants by ChIP-seq and compared them with those in *brm* single mutants. We found that removal of CLF activity led to a marked reduction of H3K27me3 levels at approximately half of the genes with increased H3K27me3 levels in *brm-1* (133 out of 258; Fig. 2B; S3 Dataset), indicating the requirement for CLF activity for the increased H3K27me3 levels at some of the genes in *brm* mutants. We validated these results by ChIP-qPCR at selected genes (Fig. 2C). It is worth noting, however, that there was no drastic loss in H3K27me3 levels at the rest of the genes in the *brm clf* double mutant relative to the *brm* single mutant (Fig. 2B and 2C), which might be explained by the redundant SWN activity at these loci. To examine the contribution of SWN, we generated the *brm-1 swn-4* double mutant. We found that H3K27me3 levels at the majority of the selected loci in the *brm swn* double mutant were lower than those in the *brm* single mutant (Fig. 2C). Furthermore, we scanned the *SVP* locus and included a region from the neighboring gene *At2g22560*, for H3K27me3 distribution in all five mutant backgrounds. As shown in Fig. 2D, the results were consistent with those in Fig. 2B and 2C, suggesting a redundant role of CLF and SWN at *SVP*. These observations are consistent with a scenario in which BRM acts to protect some gene loci from PcG activity in developing seedlings so that these genes stay active.

### Increased Occupancy of CLF/SWN at Target Loci in *brm* Mutants

**Figure 3:**
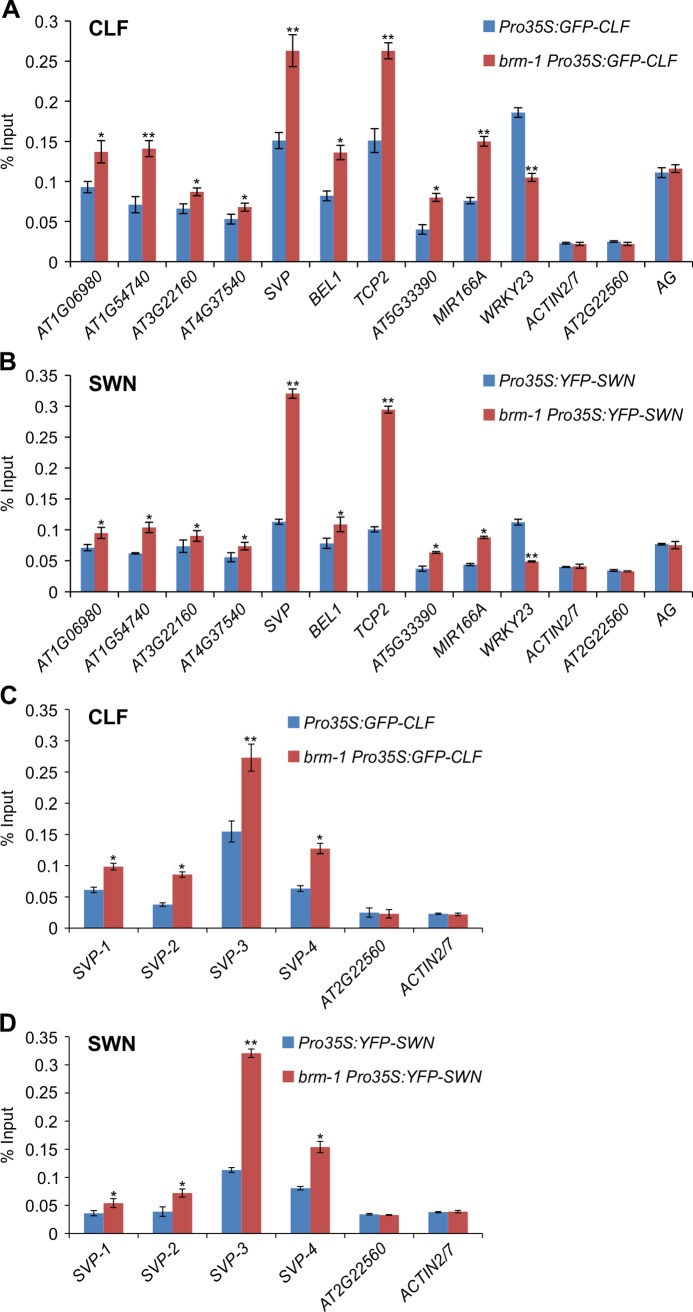
Physical occupancy of CLF and SWN at selected genes in *brm* mutants. (A) Analysis of CLF occupancy at selected genes as determined by ChIP-qPCR using anti-GFP antibody in *brm-1 Pro35S:GFP-CLF* and *Pro35S:GFP-CLF* plants. (B) Analysis of SWN occupancy at selected genes as determined by ChIP-qPCR using anti-GFP antibody in *brm-1 Pro35S:YFP-SWN* and *Pro35S:YFP-SWN* plants. (C) and (D) ChIP-qPCR to determine the levels of CLF (C) and SWN (D) occupancy across the *SVP* locus. The primers used are the same as those in Fig. 2D. ChIP signals are shown as percentage of input. *ACTIN2/7* and *AT2G22560* were used as negative control loci. *AG* was a positive control locus. Error bars indicate standard deviations among three biological replicates. *: *P* < 0.05; **: *P* < 0.01.

To determine if the increase in H3K27me3 levels in *brm* mutants was due to increased CLF/SWN presence at the loci, we first measured CLF occupancy at the loci in the *brm* mutant relative to wild-type using a GFP-tagged CLF line [43]. As shown in Fig. 3A, CLF occupancy was increased at all the selected genes, suggesting that, in the absence of BRM, CLF is allowed to access some inappropriate genomic regions, resulting in increased H3K27me3 levels. We then examined the involvement of SWN. For that, we generated an YFP-tagged SWN line and performed ChIP with anti-YFP antibodies to measure SWN occupancy in the *brm* mutant relative to wild type. We found that the occupancy of SWN was also increased at the majority of the loci when BRM was absent (Fig. 3B). Furthermore, we also scanned the *SVP* locus, including the region in the neighboring gene, to compare patterns of CLF/SWN occupancy between wild type and *brm-1*. As shown in Fig. 3C and 3D, the two proteins were markedly enriched in *brm-1* across the *SVP* locus with a strong bias towards the transcription start site (TSS), implicating a redundant action of CLF and SWN at *SVP*. These observations suggest that increased CLF/SWN occupancy could contribute to the elevated levels of H3K27me3 in *brm* mutants. On the other hand, by comparing H3K27me3 levels and CLF/SWN occupancy at *SVP* relative to the control loci such as *At2g22560* and *ACTIN*, low but significant levels of H3K27me3 (Fig. 2D) and CLF/SWN (Fig. 3C and 3D) at *SVP* were found in wild-type plants. This suggested that BRM prevents high levels of H3K27me3 and CLF/SWN occupancy rather than excluding them. Alternatively, it could also function to keep PcG in an inactive state. At the *WRKY23* locus, CLF/SWN occupancy was reduced in the *brm* mutant (Fig. 3A and 3B), consistent with the decreased H3K27me3 levels observed at this locus (Fig. 1D and 1E).

### Physical Occupancy of BRM at Target Loci

Next, we asked how BRM antagonizes PcG function during vegetative growth, i.e., whether it does so directly or indirectly. One of the possibilities that could explain the increased H3K27me3 deposition and PcG protein occupancy on chromatin in *brm* is the elevated expression level of genes encoding PcG subunits. To address this issue, we examined the expression levels of genes encoding PRC2 components, including *CLF, SWN, EMBRYONIC FLOWER2* (*EMF2*), *VERNALIZATION2* (*VRN2*), FERTILIZATION-INDEPENDENT ENDOSPERM (*FIE*) and *FERTILIZATION INDEPENDENT SEED2* (*FIS2*) [12] in *brm* mutants. The expression of these genes was not increased markedly in *brm-1* compared to that in wild-type (S4 Fig.), indicating that BRM does not antagonize PcG through repressing the expression of PcG-encoding genes. We also measured histone H3 levels at selected genes, and found a slight increase in *brm-1* (S5 Fig.). However, the change in H3 levels was very small and thus could not fully account for the change in H3K27me3 levels. These observations point to the possibility that BRM acts directly at the target loci to antagonize PcG proteins.

**Figure 4:**
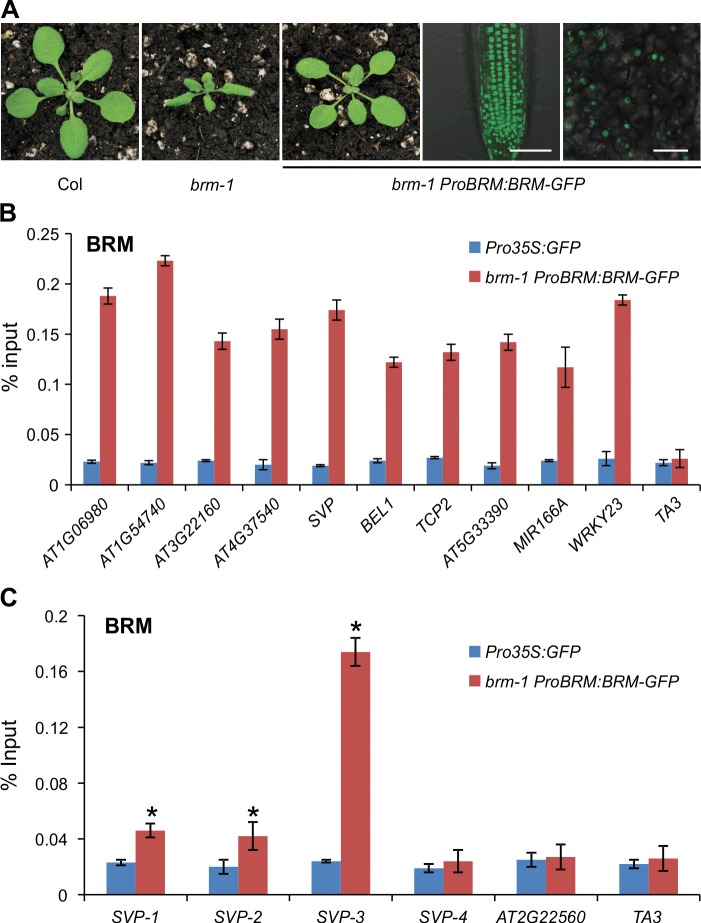
Physical occupancy of BRM at selected genes. (A) *ProBRM:BRM-GFP* could complement the *brm-1* phenotype. GFP signals were detected by confocal microscopy in 14-day-old *brm-1 ProBRM:BRM-GFP* roots and leaves, respectively. Scale bar: 50 μm. (B) BRM occupancy at selected genes as determined by ChIP using anti-GFP antibody in *brm-1 ProBRM:BRM-GFP* plants with *Pro35S:GFP* plants as control. ChIP signals are shown as percentage of input. *TA3*, a transposable element gene that is not targeted by BRM [34], was used as a negative control locus. (C) ChIP-qPCR to determine the occupancy of BRM across the *SVP* locus. ChIP signals are shown as percentage of input. The position of primer pairs used is the same as in Fig. 2D. *AT2G22560*, a flanking locus of *SVP*, was used as a negative control locus. Error bars indicate standard deviations among three biological replicates. *: *P* < 0.05.

We then tested whether BRM acts directly on the affected genes by physically binding to these genes. We performed ChIP-qPCR experiments to examine BRM occupancy at the affected genes. For the ChIP assay, we used a transgenic *Arabidopsis* line expressing a GFP-tagged BRM transgene under the control of the *BRM* native promoter (*ProBRM:BRM-GFP*) [47]. The transgene could fully rescue the morphological defects of the *brm-1* null mutant (Fig. 4A), suggesting that it is functional in vivo. ChIP was performed with anti-GFP antibodies and *Pro35S:GFP* plants were used as the negative control. The ChIP DNA was analyzed by qPCR to examine the enrichment of BRM at target genes. Genomic regions around the transcription start site at all examined genes were significantly enriched in the BRM-GFP ChIP (Fig. 4B). Furthermore, we scanned the *SVP* locus, including the negative control region in the neighboring gene, for BRM occupancy. As shown in Fig. 4C, the BRM was found to be significantly enriched at the *SVP* locus, particularly near the TSS. The physical association of BRM with these selected genes, in combination with increased H3K27me3 deposition and decreased expression of the genes in *brm* mutants, suggests that BRM acts directly at these target loci, to keep the PRC2 activity off and thus promote gene activity. Loss of BRM activity allows the access to these loci by PRC2, which turns off or decreases gene expression.

### BRM Positively Regulates *SVP* Expression

**Figure 5:**
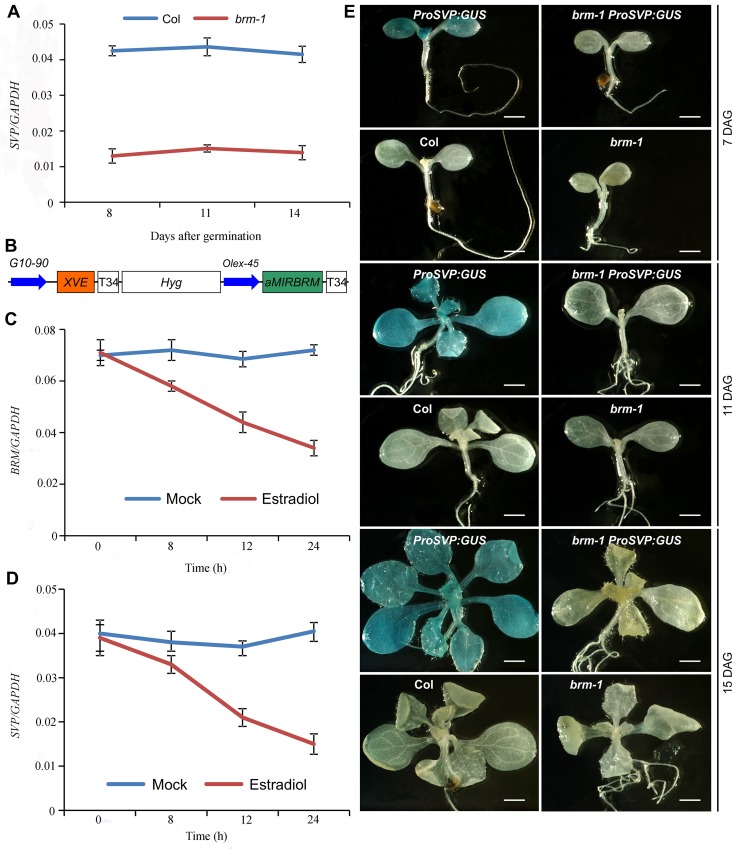
*SVP* expression is tightly controlled by BRM. (A) The expression of *SVP* is drastically decreased in developing *brm-1* seedlings compared with that in Col (grown at 22°C) as determined by qRT-PCR. (B) Schematic diagram of the region between the right and left T-DNA borders of the *XVE::aMIRBRM* construct. The precursor of *aMIRBRM* was inserted behind a LexA operator sequence fused to the-45 35S minimal promoter (O_LexA_-45). Other components of the vector were described previously (Curtis and Grossniklaus 2003). (C) *BRM* expression in 7-old-day *XVE::aMIRBRM* transgenic seedlings mock treated or treated with 10μm β-estradiol for 0, 8, 12, and 24h, respectively. (D) *SVP* expression in 7-day-old *XVE::aMIRBRM* transgenic seedlings mock treated or treated with 10μm β-estradiol for 0, 8, 12, and 24h, respectively. The expression of each gene in A, C, and D was normalized to that of *GAPDH*. Error bars indicate standard deviations among three technical replicates from one representative experiment. (E) GUS activity patterns of *ProSVP:GUS* in Col and *brm-1* backgrounds in 7, 11, and 14-DAG (days after germination) seedlings. Col and *brm-1* were included as negative controls. Scale bar: 0.5 mm.

In the sections below, we present our observations to demonstrate that *SVP* is a main target of BRM in the control of flowering. *SVP* is a key negative regulator of flowering in *Arabidopsis*, and loss-of-function of *SVP* results in early flowering [5,7]. Consistent with its role in maintaining the duration of the vegetative phase, *SVP* is highly expressed in seedlings, but is barely detectable in inflorescence tissues [7]. We noticed initially from our ChIP-seq and ChIP-qPCR data (Fig. 1D and 1E) that H3K27me3 levels drastically increased at the *SVP* locus in *brm-1* compared with wild-type. These data suggest that the *SVP* locus becomes a PRC2 target in the absence of BRM activity. The increase in H3K27me3 levels at the *SVP* locus in *brm* raises the possibility that BRM may act to keep *SVP* on by antagonizing PcG activity during vegetative growth. To test this hypothesis, we first extended the single time point expression analysis of *SVP* in *brm-1* as presented in Fig. 1F by examining the expression of *SVP* in the *brm-1* mutant spanning several developmental time points. Indeed, the expression of *SVP* in the *brm-1* mutant was consistently lower than that in wild-type plants over a time course spanning 8 to 14 days after germination (DAG, Fig. 5A), suggesting that BRM activity is continually required for the high levels of *SVP* expression in seedlings. The decreased expression of *SVP* was unlikely due to the accelerated floral transition of *brm-1* plants, since the expression of *AP1*, a marker gene for the vegetative-to-floral developmental transition [48,49], remained low throughout the time course (S6 Fig.).

To confirm that BRM activates the expression of *SVP*, we generated *XVE::aMIRBRM* transgenic lines that harbor an inducible artificial microRNA (amiRNA) targeting *BRM* (Fig. 5B). As shown in Fig. 5C, *BRM* transcript levels in 7-day-old *XVE::aMIRBRM* seedlings treated with β-estradiol to induce the amiRNA were gradually decreased by approximately 50% during a 24h time course, indicating that the amiRNA was effective. *SVP* transcript levels showed a similar reduction kinetics in the time course (Fig. 5D). This result reveals that proper BRM activity is required for *SVP* expression.

To further verify that BRM activates *SVP* expression at the transcriptional level, we obtained a previously developed *SVP* promoter-GUS fusion reporter line (*ProSVP:GUS*) [5], and introduced it into the *brm-1* background by genetic crosses (*brm-1 ProSVP:GUS*). As shown in Fig. 5E, GUS activity in *brm-1 ProSVP:GUS* was almost invisible compared to that in *ProSVP:GUS* at all three time points (Fig. 5E), suggesting that the promoter of *SVP* has no detectable activity when BRM is absent. As negative controls, we also stained Col wild-type and *brm-1* mutants but saw no signals (Fig. 5E). Documented *Arabidopsis* gene expression data indicate a temporal and spatial overlap of the *SVP* and *BRM* expression patterns in leaves (S7 Fig.) [50], which is consistent with a role for BRM as a positive regulator of *SVP* in developing seedlings. These observations demonstrate a positive spatial and temporal correlation between *BRM* and *SVP* expression, and when combined with our BRM-GFP ChIP data (Fig. 4B and 4C) that showed a direct BRM binding to the *SVP* locus, indicate that BRM directly promotes *SVP* expression during vegetative development.

### BRM Represses Flowering Mainly via Regulating *SVP* Transcription

**Figure 6:**
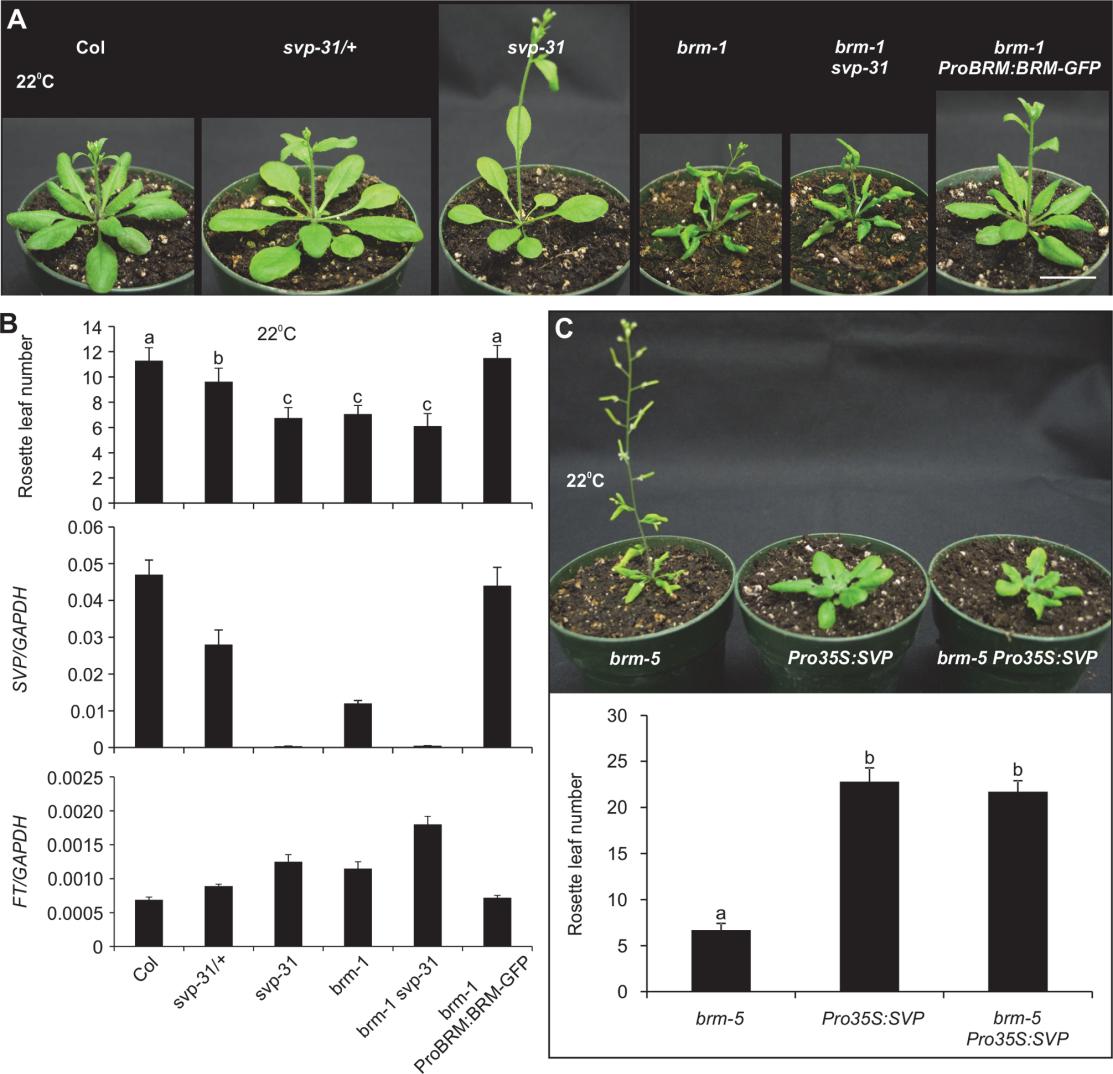
BRM represses flowering mainly through regulating *SVP* transcription. (A) Comparison of flowering phenotypes of plants with various genetic backgrounds shortly after bolting. For direct comparison, pictures of wild-type and *brm-1 ProBRM:BRM-GFP, svp-31* /+ (heterozygous) and *svp-31*, and *brm-1* and *brm-1 svp-31* were taken at the same age, respectively. All plants were grown at 22°C under long-day conditions. Scale bar: 2 cm. (B) Reduction of *SVP* expression is associated with the early flowering of *brm-1* at 22°C. Top panel: rosette leaf number at bolting of plants in different genetic backgrounds. Error bar indicates standard deviations from at least 20 plants. Lowercase letters indicate significant differences between genetic backgrounds, as determined by Post-hoc Tukey’s HSD test. Middle panel: expression analysis of *SVP*. Bottom panel: expression analysis of *FT*. The expression of *SVP* and *FT* was calculated relative to that of *GAPDH*. Error bars indicate standard deviations among three technical replicates from one representative experiment. (C) Overexpression of *SVP* rescues the early flowering phenotype of *brm* mutant. Top panel: flowering phenotype of *brm-5, Pro35S:SVP* and *brm-5 Pro35S:SVP* plants grown for five weeks at 22°C under long-day conditions. Scale bar: 2 cm. Bottom panel: rosette leaf number of *brm-5, Pro35S:SVP* and *brm-5 Pro35S:SVP* plants at bolting. Lowercase letters indicate significant differences between genetic backgrounds, as determined by Post-hoc Tukey’s HSD test.

Having shown above a positive role for BRM in regulating *SVP* expression, we next sought to investigate whether the BRM-SVP module can largely explain the early flowering phenotype of the *brm* mutant. Both *brm* and *svp* single mutants show early flowering phenotypes under long-day conditions [6,7,33,39,40], but it is not known whether there is a common molecular mechanism underlying their flowering phenotypes. We first estimated the flowering time in the two mutants by counting the number of leaves at bolting (Fig. 6A and 6B, top panel). *brm-1* and *svp-31*, a null T-DNA insertion mutant [6], flowered at roughly the same time. The *svp-31* heterozygous plants flowered significantly later than their homozygous siblings but earlier than wild-type Col plants, indicating that SVP controls flowering in a dosage-dependent manner, consistent with previous observations [7]. Next, taking advantage of the dosage-dependent nature of flowering control by SVP, we quantified *SVP* transcript levels by qRT-PCR in the mutant plants to estimate the contribution of SVP to the flowering control by BRM (Fig. 6B, middle panel). Our qRT-PCR data confirmed that *svp-31* is a null allele and the heterozygous plants accumulated approximately half the amount of *SVP* transcripts found in wild-type plants (Fig. 6B, middle panel). *SVP* expression in *brm-1* was drastically decreased to less than half that of *svp-31* heterozygous plants. In *brm-1 ProBRM:BRM-GFP* plants, both the flowering time and *SVP* expression were restored to the wild-type level (Fig. 6A and 6B), further confirming that BRM activity is responsible for the normal expression level of *SVP*. Our quantification of flowering time and *SVP* transcript levels in *brm-1*, when compared quantitatively to those from *svp-31* mutants, suggests that 1) BRM is a major activator of *SVP* expression; and 2) The early flowering phenotype of the *brm-1* mutant can largely be accounted for by the down regulation of *SVP* transcription in the mutant, although other BRM targets also have minor contributions.

To provide additional evidence to strengthen our conclusion, we tested whether restoration of *SVP* in *brm* mutant background could overcome its early flowering phenotype by expressing *SVP* from a promoter that is not controlled by BRM (*Pro35S:SVP*) [51] in *brm*-*5*, a chemically induced mutant that has a single nucleotide change in the region encoding the ATPase domain [33]. Indeed, introduction of *Pro35S:SVP* into *brm-5* could rescue the early flowering of the *brm-5* mutant (Fig. 6C). We also generated a *brm-1 svp-31* double mutant to test the genetic interaction between *BRM* and *SVP* in flowering time control. The *brm-1 svp-31* double mutant flowered only slightly earlier than either single mutant (Fig. 6B), suggesting that BRM and SVP act largely in a common pathway in determining flowering time, and only minor contributions from other BRM targets. It is worth mentioning that three other flowering time genes also displayed increased H3K27me3 levels in the *brm* mutant (S2 Dataset and S8 Fig.). When we checked the expression of these genes, we only saw a clear decrease of *AGAMOUS-LIKE24* (*AGL24*) expression but not the other two in *brm-1* (S8 Fig.). AGL24 is a MADS-box protein involved in flowering time control. *agl24* mutants show delayed flowering while *agl24 svp* double mutants are early flowering as *svp* single mutants [52]. The data thus suggest that the early flowering phenotype of *brm* mutants is unlikely caused by these flowering time genes. In addition, we also examined the expression of *FT*, a well-established SVP target, in the various genetic backgrounds (Fig. 6B, bottom panel). As expected, *FT* transcript levels correlated negatively with those of *SVP* and positively with flowering time in the corresponding genetic backgrounds. In summary, our observations strongly suggest that BRM represses flowering mainly through activating *SVP*.

## Discussion

In both animals and plants, a group of proteins that counteract PcG function have been described and referred to as TrxG proteins [9,13]. Several putative TrxG proteins have been proposed in *Arabidopsis*, including the H3K4 methyltransferase ATX1 [53], the SAND-domain DNA binding protein ULTRAPETALA1 (ULT1) [54], the chromatin remodeling ATPase PICKLE (PKL) [55], the H3K27me3 demethylase REF6 [19], and the SWI2/SNF2 ATPases SPLAYED (SYD) and BRM [34]. A challenge for the field is to understand the specific roles of the putative TrxG proteins and the functional relationship among them in antagonizing PcG.

**Figure 7:**
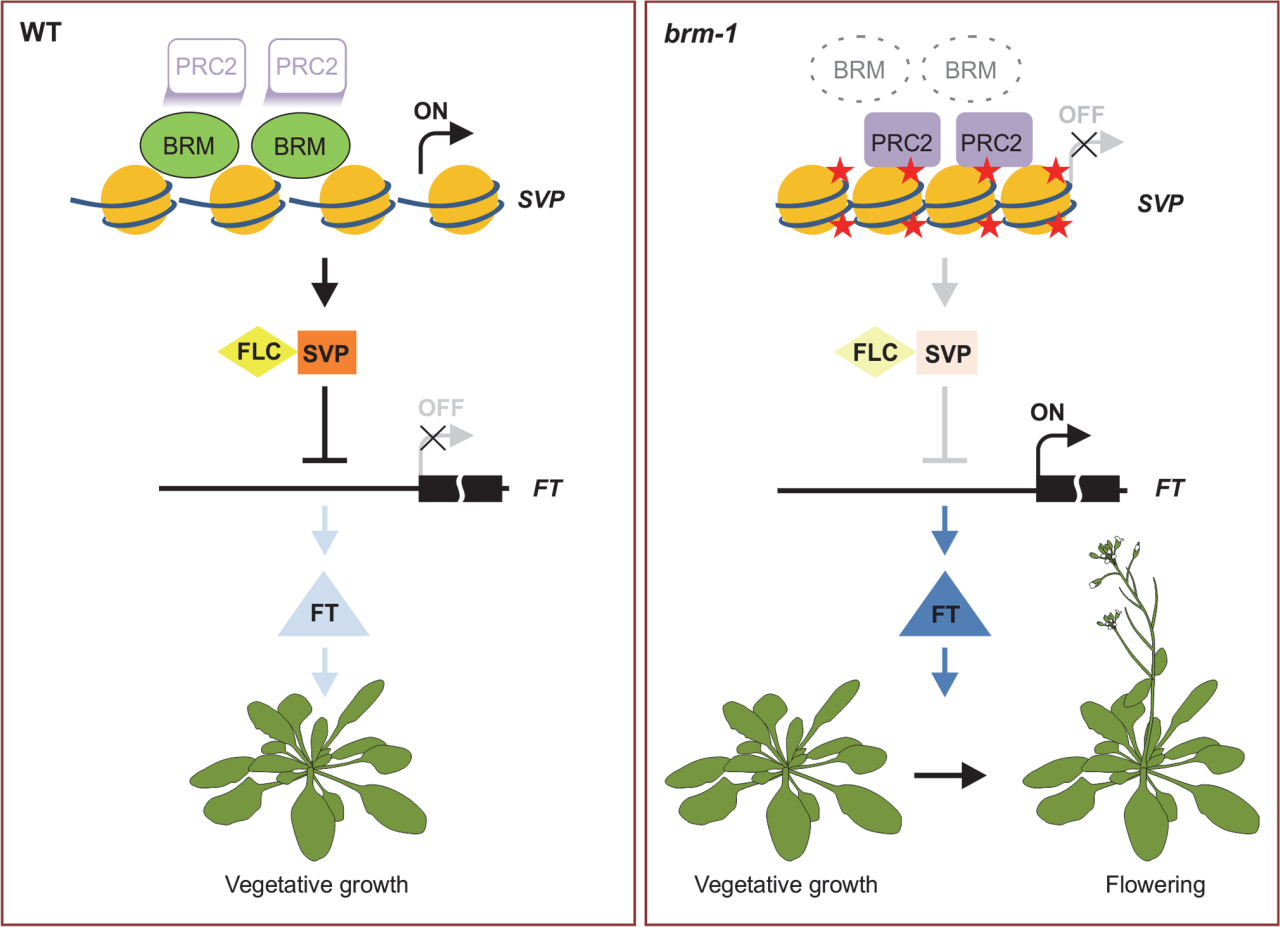
A model for BRM in preventing inappropriate PcG activities at *SVP* to promote vegetative growth. In wild-type plants, BRM is physically present at the target chromatin sites, and suppresses the inappropriate silencing of target genes by PcG, thus maintaining the expression of the target loci where such expression is needed at the specific developmental phase. Without BRM, PcG is allowed access to some inappropriate genomic sites, resulting in increased H3K37me3 levels and consequently down-regulation of the expression of the genes. For example, *SVP* is highly expressed in wild-type seedlings and its downstream target *FT* is repressed, therefore, vegetative growth is promoted. Conversely, *SVP* expression is repressed by the mistargeting of PRC2 in *brm* mutants, and *FT* is de-repressed as a result to lead to early flowering. Red star: the H3K27me3 mark.

The nature of the antagonism between SWI/SNF-type chromatin remodeling ATPases and PcG proteins has been investigated in several studies in animals; and several models of counteraction have been proposed [23,25,56–58]. Interestingly, a very recent report in *Arabidopsis* showed that BRM overcomes the repression of *AG* and *AP3* by the PcG pathway during the initiation of floral development [34], however, how it does so and to what extent BRM is required for antagonizing PcG function in plants remains unclear. Our genome-wide study shows that BRM deficiency led to an increase in H3K27me3 levels at several hundred genes across the genome during vegetative development in *Arabidopsis*. We further observed increased occupancy of CLF/SWN-containing PcG complex(es) at these genes when BRM is not located there (Fig. 3; S1 Table). Considering that there are low but significant levels of H3K27me3 (Fig. 2B–2D) and CLF/SWN occupancy (Fig. 3) at *SVP* in wild-type plants, we favour a model of antagonism between BRM and PcG, in which BRM might function to prevent high levels H3K27me3 and CLF/SWN occupancy instead of excluding them (Fig. 7). It is also possible that BRM could function to keep PcG in an inactive state. In addition to chromatin remodelers, plants might employ transcription factors to counteract PcG. A recent study showed that the binding of transcription factor AG to the promoter of zinc finger repressor *KNUCKLES* (*KNU*) causes the eviction of the PcG proteins from the locus, leading to the induction of *KNU* [59]. Thus, both transcription factors and chromatin remodeling proteins could be involved in counteracting PcG. It will be interesting to determine whether and how these two machineries work together in antagonizing PcG function.

Our genome-wide analysis of H3K27me3 occupancy in *brm* mutant indicates that BRM does not only antagonize PcG function during plant development, but also cooperates with PcG at some loci (Fig. 1D and 1E). For example, the H3K27me3 level at *WRKY23* is decreased and the expression of the gene is up-regulated in both *brm* and *fie* mutant (FIE is a PcG subunit) seedlings [17] (this study), suggesting that both BRM and PcG are required for the proper expression of *WRKY2*3. Further, we show that the decreased H3K27me3 observed at *WRKY23* loci in *brm* mutant could be because of, at least partly, the decreased CLF binding. Therefore, this observation suggests that BRM may work with the PcG proteins at some of the common loci and thus repress the targets expression. WRKY23 was recently found to be needed for proper root development and the over-expression of *WRKY23* results in the reduction of root length [60]. It will be interesting to test whether the increased transcription of *WRKY23* could explain the short root phenotype of *brm* [28]. The synergistic relationship between BRM and PcG reported here was also observed by a study in human embryonic stem cell showing that an embryonic stem cell specific SWI/SNF complex acts synergistically with PRC2 at all four *Hox* loci [25]. The mechanism by which BRM cooperates with PcG is currently unknown. One possibility would be that BRM directly interacts with PcG and facilitates the targeting of PcG to genes. Indeed, we found that BRM co-localizes with H3K27me3 at the *WRKY23* locus in wild-type seedlings (Fig. 4), suggesting that BRM might interact with PcG proteins. However, no study so far has demonstrated a direct physical interaction between BRM and PcG proteins. It is possible that these two complexes might interact transiently or indirectly. Nevertheless, the synergistic relationship between BRM and PcG found in both animals and plants might suggest its biological relevance and warrants further studies.

The proper transition from vegetative growth to flowering is critical for the reproductive success of angiosperm plants and must be controlled precisely. BRM has been proposed as a repressor of flowering as suggested by the early flowering phenotype and the elevated *FT* expression of *brm* mutants [39,40]. However, it was not clear whether BRM acts directly or indirectly to repress *FT. SVP* has been demonstrated to be a direct repressor of *FT* [5,6], and thus serves as a key repressor of floral transition. The precise regulation of *SVP* is obviously of critical importance for our understanding of flowering control. Thus far, however, no direct upstream activator of *SVP* has been identified. In this work, we provide evidence demonstrating that BRM represses *FT* by directly maintaining a high level of *SVP* expression (Fig. 7). First, loss of BRM activity results in decreased expression of *SVP* (Fig. 5A–5E), which is associated with increased H3K27me3 levels (Fig. 1D and 1E) and increased occupancy of CLF and SWN (Fig. 3C and 3D). Second, BRM directly binds to the *SVP* locus in vegetative tissues where *SVP* is highly expressed (Fig. 4B and 4C). Together, these observations suggest that BRM represses the floral transition through directly activating *SVP*. This is consistent with the genetic evidence showing that the *brm-1 svp-31* double mutant displays almost the same early flowering phenotype as *brm-1* and *svp-31* single mutants (Fig. 6A and 6B). Although our data support a scenario that BRM represses flowering mainly through *SVP*, some evidence suggests that BRM may also repress flowering through other pathways. For example, the expression of *CONSTANS* (*CO*), an activator of *FT* in the photoperiod pathway, was increased in *brm* mutants [40]. In addition, elevated expression of both *FLC* and *FT* in *brm* mutants was also reported previously [39,40]. Since *FLC* is a repressor of *FT* expression [61], it seems hard to understand why the expression levels of both *FLC* and *FT* were increased in *brm* mutants. Our results presented here provide an explanation for this apparent discrepancy: mutation of *BRM* results in reduced expression of *SVP* and consequently lower abundance of the SVP-FLC repressor complex, ultimately leading to activation of *FT*, regardless of the increased expression of *FLC*.

It is also relevant to note that down-regulation of *BAF60/SWP73B* was recently reported to cause increased *FLC* expression and delayed floral transition [37]. The apparently opposing flowering time phenotype of *brm* mutants and the *BAF60* knockdown line is puzzling. It is unknown whether and how BAF60 regulates *SVP* expression. It might be possible that the presence of BAF60 in a SWI/SNF complex inhibits the activity of BRM, thus reduction of BAF60 could allow BRM to activate *SVP* expression, which, in turn, leads to delayed floral transition. Alternatively, it might also be possible that BRM and BAF60 are present in distinct complexes that differ in their regulatory activities and target genes, e.g., BRM activates *SVP*, while BAF60 represses *FLC*.

Our genome-wide H3K27me3 profiling data also reveal that BRM is involved in the regulation of a number of other important developmental genes including, most noticeably, members of the *miR166* and *miR156* families (S2 Dataset). It is well established that the *miR166* family miRNAs target the transcripts of the HD-ZIPIII genes, controlling the level and domain of their expression to allow their proper functions in plant development [62–64]. More recently, we uncovered a new role for *miR166* in repressing the seed maturation program during vegetative development [65]. An earlier study demonstrated the involvement of BRM in repression of the seed maturation genes in leaves [33] – a *brm* mutation was isolated in a reporter-assisted genetic screen for *Arabidopsis* mutants exhibiting ectopic expression of seed storage protein genes [33,65,66]. Our new data presented here thus provide a potential link between the two early studies [33,65]: it strongly suggests that BRM promotes the accumulation of *miR166*, which in turn represses seed maturation genes in developing seedlings. In conclusion, our work demonstrates that BRM promotes vegetative development by harnessing PcG proteins (mainly by preventing their activities) at key developmental genes.

## Materials and Methods

### Plant Materials and Growth Condition

*Arabidopsis* seeds were stratified at 4°C for 3 days in dark condition. Then the seeds were sown on soil or on agar plates containing 4.3 g/L Murashige and Skoog nutrient mix (Sigma-Aldrich), 1.5% sucrose, 0.5 g/L MES (pH 5.8), and 0.8% agar. Plants were grown in growth rooms with 16-h-light/8-h-dark cycles (Long-day, LD) at 22°C or 16°C. T-DNA insertion mutants were obtained from the ABRC, unless otherwise indicated. The *brm-1* (SALK_030046), *brm-5, clf-29* (SALK_021003), *tfl2-1* (CS3796), *svp-31* (SALK_026551) and *SWN-4* (SALK_109121) mutants are all in the Col background and have been described previously [6,28,33,46,67,68]. Homozygous T-DNA insertion mutants were identified by PCR-based genotyping. Transgenic plants *ProBRM:BRM-GFP, ProSVP:GUS, Pro35S:SVP, Pro35S:GFP-CLF* and *Pro35S:GFP* have been described [5,43,47,69].

### Chromatin Immunoprecipitation (ChIP) Assays

ChIP was carried out as described [70,71] with minor modifications. Briefly, two grams of 14-day-old seedlings grown on MS agar were cross-linked with 1% formaldehyde and then ground into fine power with liquid nitrogen. Chromatin was isolated and sheared into 200–800 base pair fragments by sonication. The sonicated chromatin was immunoprecipitated with 5 μL of anti-H3K27me3 (07–449, Millipore), anti-GFP (ab290, Abcam) or anti-H3 (Ab1791, Abcam) antibodies. The precipitated DNA was then recovered with the MiniElute PCR Purification Kit (Cat#28004, Qiagen) according to the manufacturer’s instructions. ChIP-qPCR was performed with three technical replicates, and results were calculated as percentage of input DNA according to the Champion ChIP-qPCR user manual (SABioscience). If fold enrichment was used, the calculated percentage input of the wild-type control plant at the regions tested was set to 1. The fold enrichment represents the fold change relative to the wild-type. Independent ChIP experiments were performed at least two more times and similar results were obtained. Primer sequences used for ChIP-qPCR were listed in S2 Table.

### ChIP-seq Analysis

Ten ng of ChIP DNA immuoprecipitated by the anti-H3K27me3 antibody was used for ChIP-seq library construction. End repair, adapter ligation and amplification were carried out using the Illumina Genomic DNA Sample Prep Kit according to the manufacturer’s protocol. Illumina Genome Analyser IIx or HiSeq 2500 was used for high-throughput sequencing of the ChIP-seq library. The raw sequence data were processed using the Illumina sequence data analysis pipeline GAPipeline1.3.2. Then Bowtie [72] was employed to map the reads to the *Arabidopsis* genome (TAIR10) [73]. Only perfectly and uniquely mapped reads were retained for further analysis. Then the data were analyzed as described [19]. Briefly, the alignments were first converted to WIG files using MACS [74]. Then the data were imported to Integrated Genome Browser (IGB) [75] for visualization. Secondly, the program SICER [76] was used to identify ChIP-enriched domains (peaks) in histone modification signals. Thirdly, quantitative comparisons between wild-type Col and mutants were performed using the ChIPDiff program [77]. Regions with more than twofold changes were kept for further analysis. Finally, the identified regions were annotated according to the Arabidopsis annotation gff file (TAIR10, www.arabidopsis.org) using a customized Perl script. Two independent biological replicates were used for sequencing, and only the regions of H3K27me3 found in both replicates were included in the analyses.

### Gene Expression Analysis

Total RNA was isolated from ∼100 mg of plant tissues using the RNeasy Plant Mini kit (Qiagen). One μg RNA was reverse transcribed into cDNA using the High Capacity cDNA Reverse Transcription kit (ABI). Random primers from the kit were used as primers. Real-time quantitative PCR was conducted using the SsoFast EvaGreen Supermix kit with the Bio-Rad CFX96 real-time PCR detection system (Bio-Rad Laboratories, Inc.). The data shown in the figures are the average of three technical replicates. Results were repeated with two additional independent RNA samples (biological replicates). *GAPDH* served as the internal reference. PCR primers used in real-time PCR are listed in S2 Table.

For genome-wide expression analysis, three biological replicates of Col, *brm-1, clf-29* and *brm-1 clf-29* samples were analyzed on Affymetrix ATH1 arrays. Genes showing 1.5 fold changes were considered to be differentially expressed.

### Construction of YFP-SWN Transgenic Line

The *SWN* gene without the stop codon was amplified by PCR and cloned into the pDONR221 vector (Invitrogen) by BP reaction according to the manufacturer’s instructions. The resulting transgene in the entry vector was sequenced to make sure that no mutation was introduced during PCR. The transgene was then transferred into the pEarlyGate 104 Gateway-compatible destination vector [78] by LR reaction, according to the manufacturer’s instructions, to make *Pro35S:YFP-SWN*. The construct was introduced into *Agrobacterium tumefaciens* GV3101, which was then used to transform *swn-4* mutant plants using the floral dip method [79]. Transgenic plants were selected in MS agar media containing 50 μg/ml of Hygromycin B and allowed to grow in soil to maturity to yield seeds. PCR primers used in making the construct are listed in S2 Table.

### Artificial miRNA Transgene Constructs

For generating the *XVE::aMIRBRM* construct, the pRS300 vector [80] was used as the backbone to first generate *aMIRBRM*. The primers used were designed according to WMD3 (http://wmd3.weigelworld.org/cgi-bin/webapp.cgi) and are listed in S2 Table. *aMIRBRM* was subcloned into the pDONR221 vector (Invitrogen), confirmed by sequencing, and then recombined into the pMDC7 Gateway-compatible destination vector [78] where the *aMIRBRM* transgene is controlled by a Estradiol-inducible promoter. The construct was transformed into Col wild-type plants by the floral dip method [79]. Transgenic plants were selected for Hygromycin B resistance and allowed to grow to maturity to yield seeds. Seven-day-old T2 transgenic plants were treated either by 10μmol β-Estradiol or DMSO (as the mock control) and samples were collected at different time points after the treatment.

### Histochemical GUS Staining

The standard GUS staining solution (0.5 mg/mL 5-bromo-4-chloro-3-indolyl-glucuronide, 20% methanol, 0.01 M Tris-HCl, pH 7.0) was used. Seedlings immersed in GUS staining solution were placed under vacuum for 15 min, and then incubated at 37°C overnight. The staining solution was removed and samples were cleared by sequential incubation in 75% and 95% ethanol.

### Flowering Time Measurement

Wild-type and mutant plants were grown side by side in soil at 22°C or 16°C with 16-h-light/8-h-dark cycles. The number of rosette leaves was counted when the length of the inflorescence stem was 1 cm. For each genotype, at least 20 plants were analyzed, and the analysis was repeated 3 times independently.

### Accession Numbers

Sequence data from this article can be found in the Arabidopsis Genome Initiative or GenBank/EMBL databases under the following accession numbers: *BRM* (AT2G46020), *SVP* (AT2G22540), *CLF* (AT2G23380), *BEL1* (AT5G41410), *TCP2* (AT4G18390), *WRKY23* (AT2G47260), *miR156D* (AT5G10945), *LHP1/TFL2* (AT5G17690), *EMF2* (AT5G51230), *FIE* (AT3G20740), *SWN* (AT4G02020), *VRN2* (AT4G16845), *AP1* (AT1G69120), *TA3* (AT1G37110), *ACTIN2/7* (AT5G09810), *AG* (AT4G18960), *AP3* (AT3G54340), *FLC* (AT5G10140), *AGL24* (AT4G24540), *SMZ* (AT3G54990) and *FT* (AT1G65480). All raw ChIP-seq dataset and ATH1 expression array dataset have been deposited in the Gene Expression Omnibus database under accession number GSE47202 and GSE53623.

## Supporting Information

S1 Fig Leaf curling phenotype of *brm-1, clf-29, brm-1 clf-29, brm-1 tfl2-1, and brm-1 swn-4*. (A) Top panel: Rosette leaves from 14-d-old plants are shown. Scale bar: 2 mm. Bottom panel: Percentage of upwardly curled leaves in each genetic background is shown. Error bar indicates standard deviations from at least 20 plants. *P* values were determined by two-tailed *t*-test. (B) Comparison of *brm-1* and *brm-1 swn-4* double mutants grown in soil for 14 days. Scale: 1cm.
(PDF)

S2 Fig Ectopic expression of floral homeotic genes in the *clf* mutant could be partially restored by removing BRM activity. Expression data of floral homeotic genes, *AG* (A), *AP1* (B), *AP3* (C) and *AGL24* (D), in different genetic backgrounds were determined by qRT-PCR with three biological replicates.
(PDF)

S3 Fig The number of genes showing at least 1.5 fold change in 14-d-old *brm-1, clf-29*, and *brm-1 clf-29* seedlings as determined by microarray-based gene expression profiling. (PDF)

S4 Fig Expression analysis of PcG-encoding genes in *brm-1* and col seedlings as determined by qRT-PCR. The expression levels of each gene were normalized to that of *GAPDH*, and the expression level in Col was set to 1. Error bars indicate standard deviation among three technical replicates from one representative experiment.
(PDF)

S5 Fig ChIP-qPCR analyses of histone H3 levels at selected genes in *brm-1* and Col seedlings. ChIP signals are shown as fold changes relative to that in wild-type plants. Error bars indicate standard deviation among three technical replicates from one representative experiment.
(PDF)

S6 Fig Expression analysis of *AP1* in *brm-1* and Col seedlings as determined by qRT-PCR. The expression level of *AP1* gene was normalized to that of *GAPDH*. Error bars indicate standard deviation among three technical replicates from one representative experiment.
(PDF)

S7 Fig Expression patterns of *BRM* and *SVP*. The data were extracted from Schmid et al [50] and displayed using the AtGenExpress Visualization Tool (http://jsp.weigelworld.org/expviz/expviz.jsp).
(PDF)

S8 Fig Analysis of H3K27me3 and expression levels at several flowering time Genes in *brm-1*. (A) ChIP-seq data showing an increase in H3K27me3 levels at several genes in *brm-1*. Data from the wild-type Col is shown in red at the top, and *brm-1* is shown in orange at the bottom. (B) ChIP-qPCR validation using independent samples. Data are shown as percentage of input. Error bars indicate standard deviations among three technical replicates from one representative experiment. (C) Expression analysis of *AGL24* and *SMZ* by qRT-PCR. The expression of each gene was normalized to that of *GAPDH*, and the expression level in Col was set to 1. Error bars indicate standard deviations among three technical replicates from one representative experiment. (D) Small RNA northern blot analysis of *miR156* in *brm-1* compared with Col. Two time points were used (10 days and 14 days after germination). The levels of small RNAs in *brm-1* were compared with those in Col, which was set as 1. The numbers below the gel images indicate relative abundance. U6 served as loading control. RNA isolation and hybridization for miRNA detection was performed as described [65]. Digoxigenin-labeled miRNA probes were generated using the mirVana miRNA Probe Construction Kit (Ambion). Oligonucleotide probes used are listed in S2 Table.
(PDF)

S1 Table Summary of changes in H3K27me3 levels, CLF occupancy and gene expression in *brm-1* mutants. (PDF)

S2 Table Oligonucleotides used in this study. (PDF)

S1 Dataset List of genes occupied by H3K27me3 in 14-day-old Col seedlings. (XLSX)

S2 Dataset List of genes with more than 2-fold changes in H3K27me3 levels in *brm-1* seedlings compared with Col. (XLSX)

S3 Dataset Genes at which increased H3K27me3 levels in *brm-1* are dependent on CLF. (XLSX)
