# Supplementary figures and images for: The Arabidopsis SWI2/SNF2 Chromatin Remodeler BRAHMA Regulates Polycomb Function during Vegetative Development and Directly Activates the Flowering Repressor Gene SVP
Source: PLoS Genet. 2015 Jan 23;11(1):e1004944. doi: 10.1371/journal.pgen.1004944 (PMC4304717; doi:10.1371/journal.pgen.1004944)

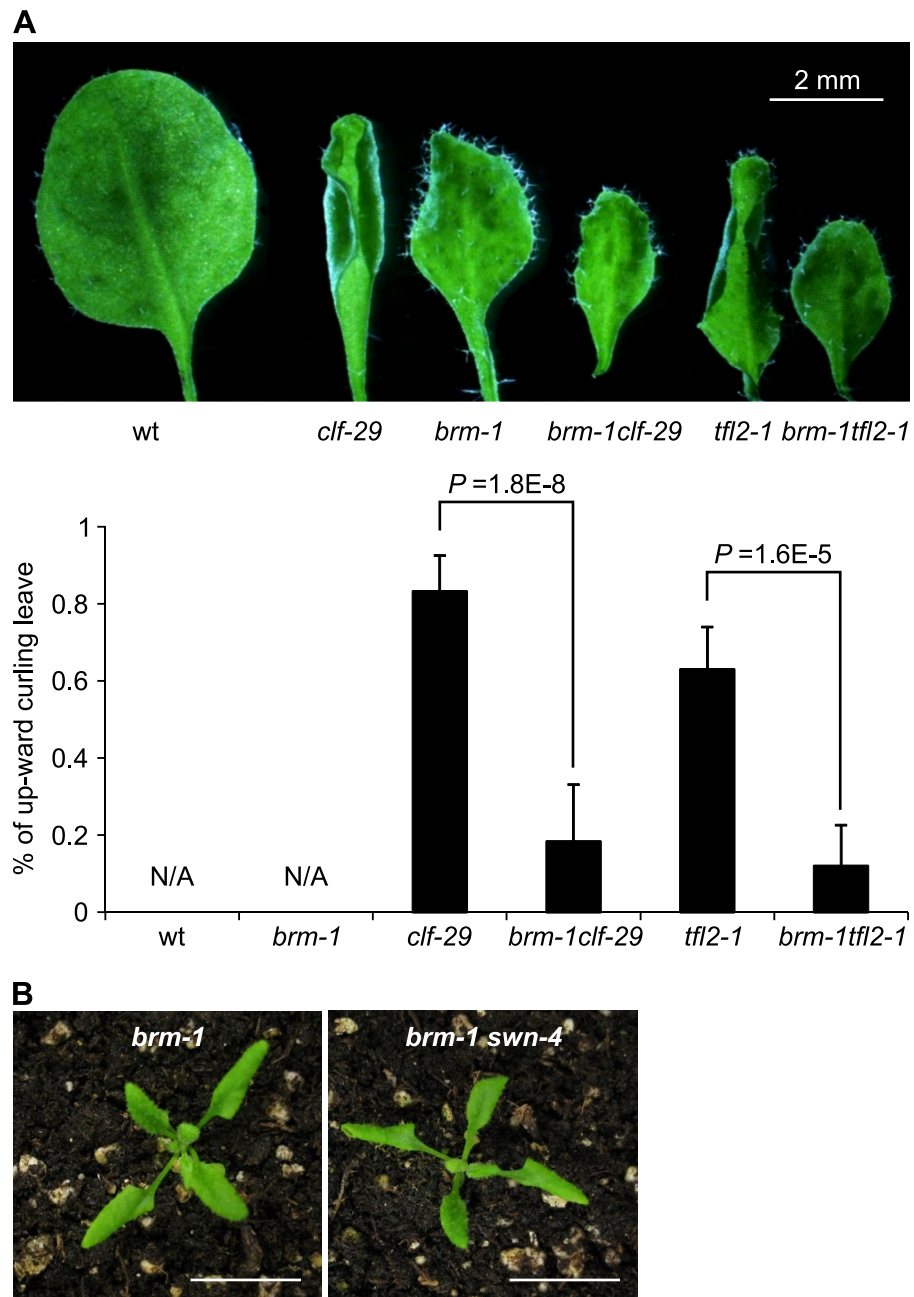

Fig S1

Supplement: S1 Fig — (A) Top panel: Rosette leaves from 14-d-old plants are shown. Scale bar: 2 mm. Bottom panel: Percentage of upwardly curled leaves in each genetic background is shown. Error bar indicates standard deviations from at least 20 plants. P values were determined by two-tailed t-test. (B) Comparison of brm-1 and brm-1 swn-4 double mutants grown in soil for 14 days. Scale: 1cm. (PDF) [file pgen.1004944.s001.pdf]

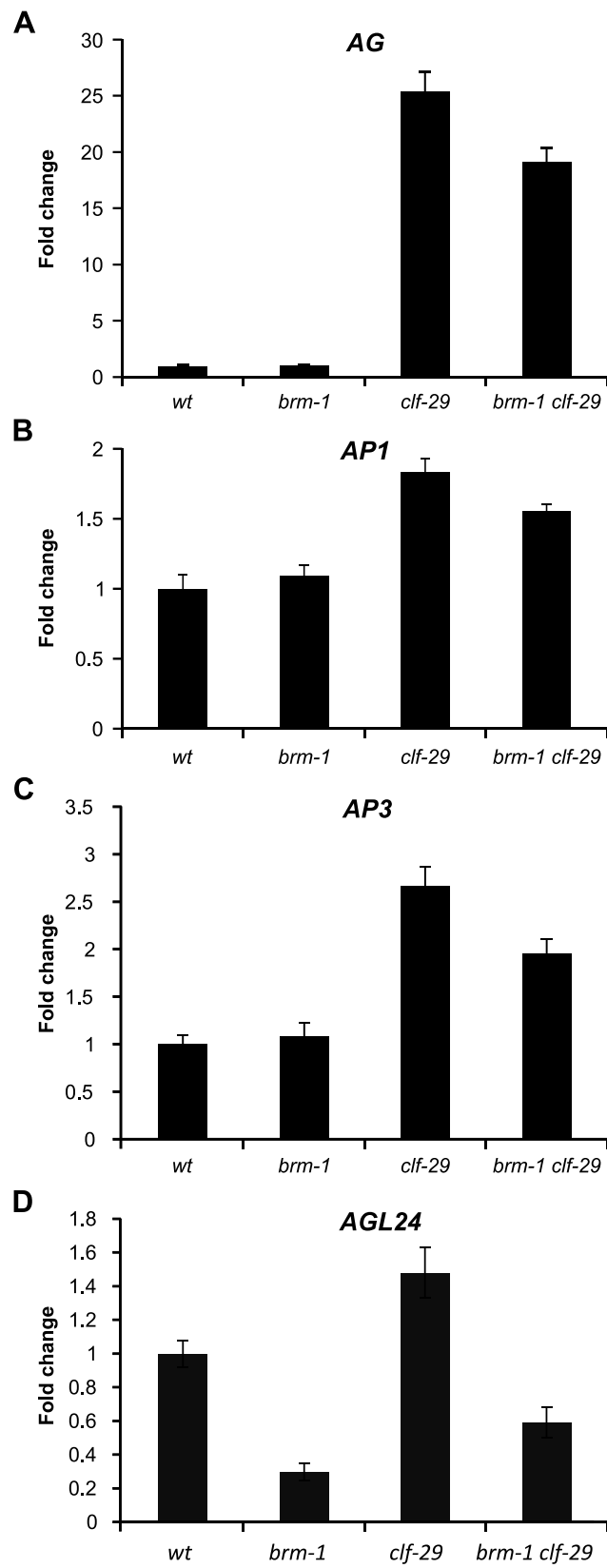

Fig S2

Supplement: S2 Fig — Expression data of floral homeotic genes, AG (A), AP1 (B), AP3 (C) and AGL24 (D), in different genetic backgrounds were determined by qRT-PCR with three biological replicates. (PDF) [file pgen.1004944.s002.pdf]

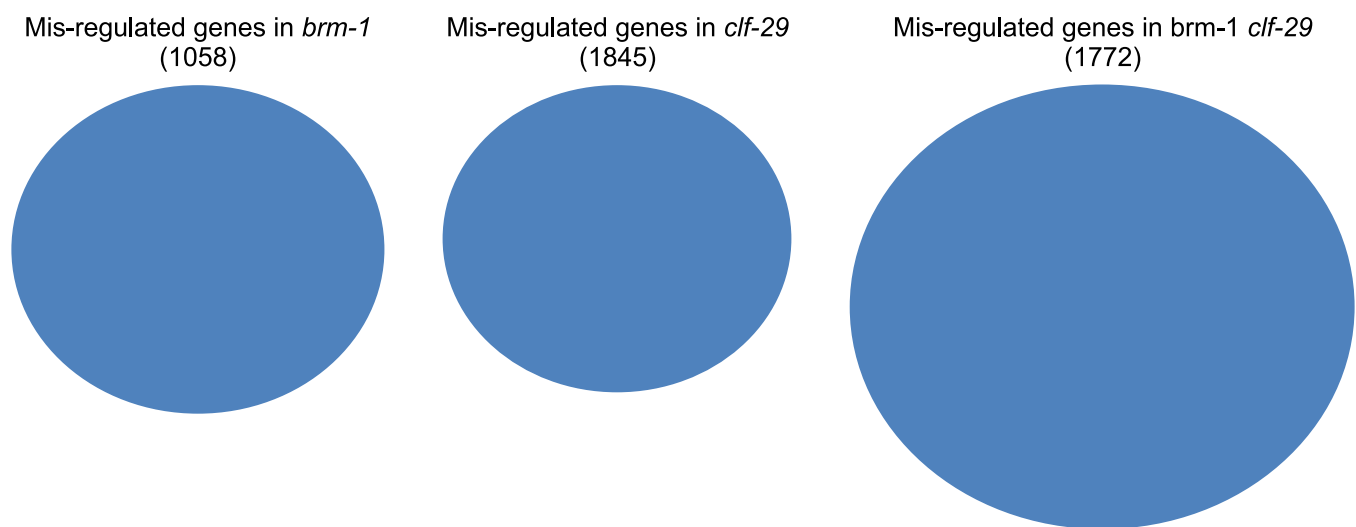

Fig S3

Supplement: S3 Fig — (PDF) [file pgen.1004944.s003.pdf]

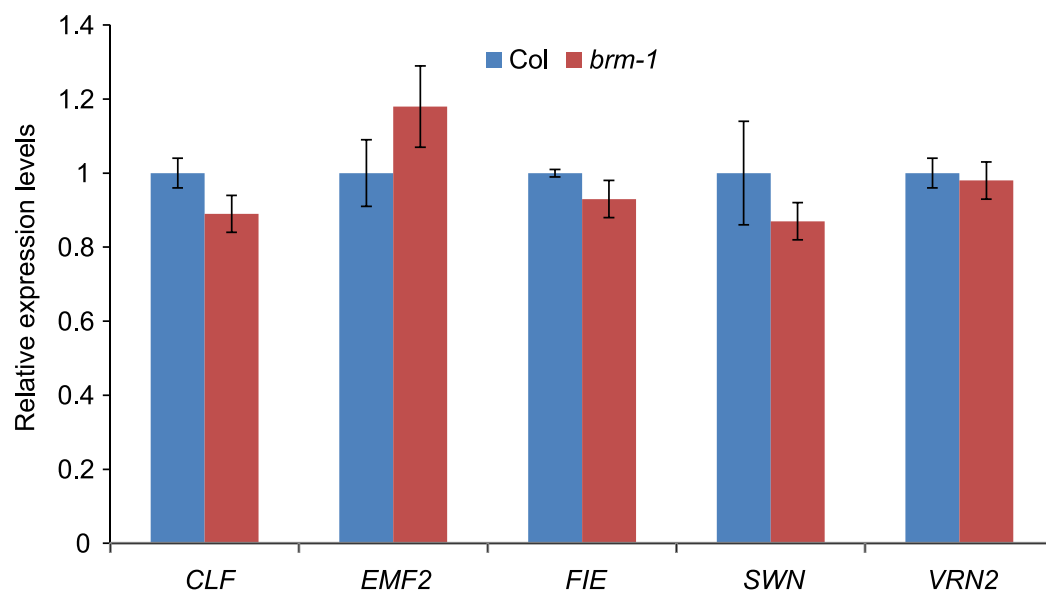

Fig S4

Supplement: S4 Fig — The expression levels of each gene were normalized to that of GAPDH, and the expression level in Col was set to 1. Error bars indicate standard deviation among three technical replicates from one representative experiment. (PDF) [file pgen.1004944.s004.pdf]

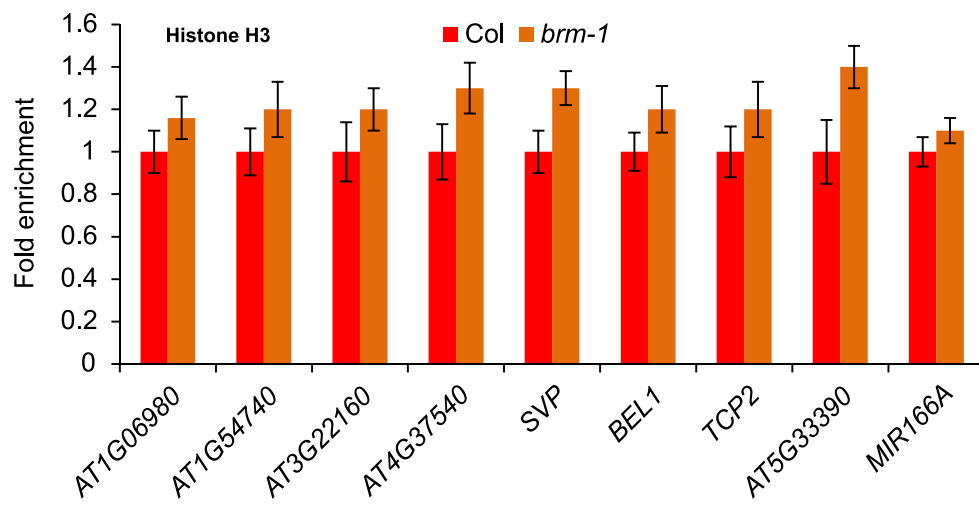

Fig S5

Supplement: S5 Fig — ChIP signals are shown as fold changes relative to that in wild-type plants. Error bars indicate standard deviation among three technical replicates from one representative experiment. (PDF) [file pgen.1004944.s005.pdf]

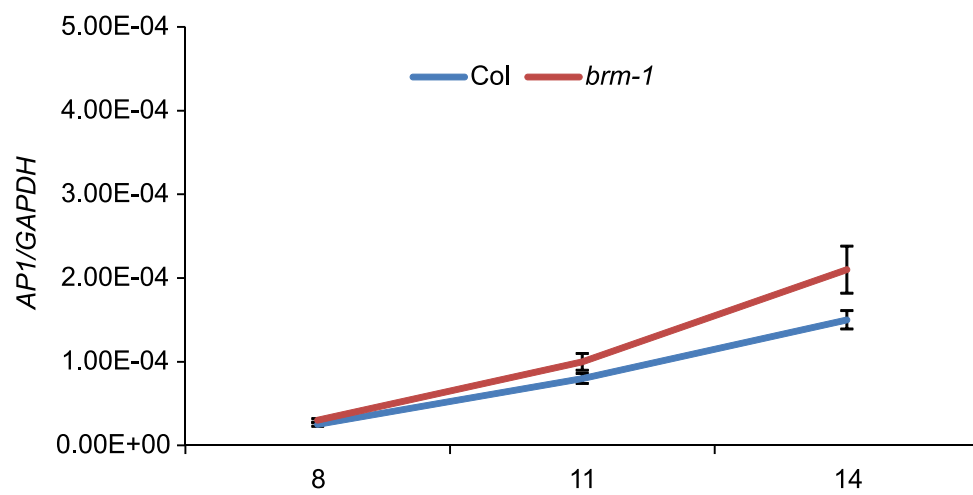

Fig S6

Supplement: S6 Fig — The expression level of AP1 gene was normalized to that of GAPDH. Error bars indicate standard deviation among three technical replicates from one representative experiment. (PDF) [file pgen.1004944.s006.pdf]

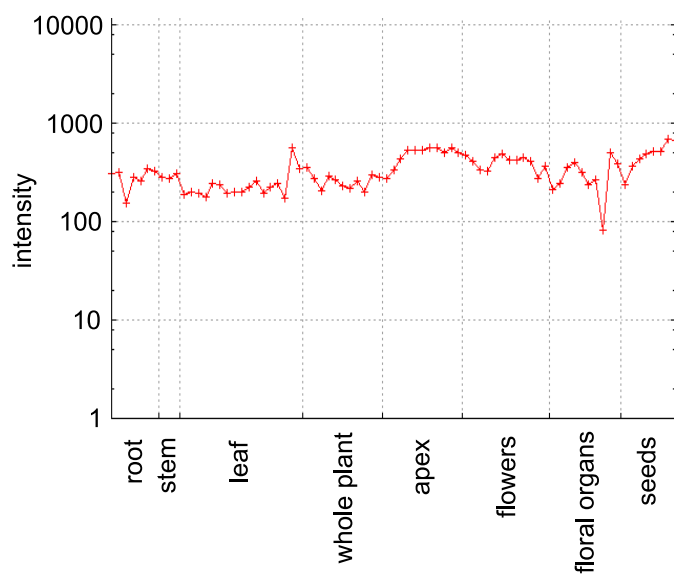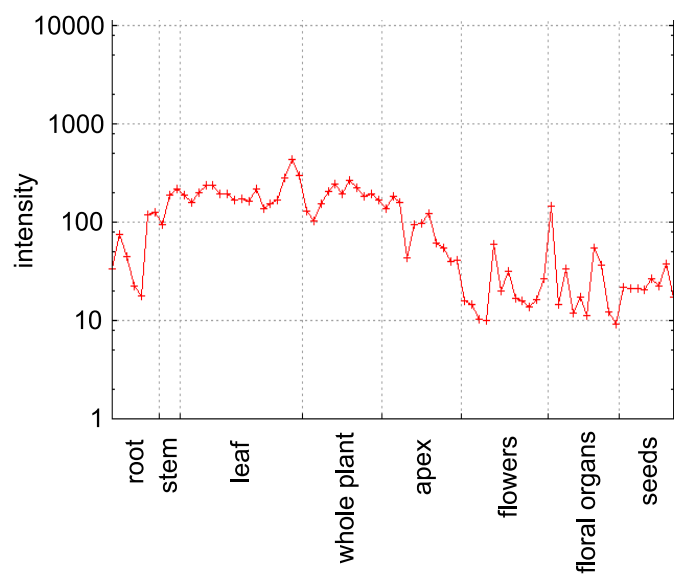

Fig S7

Supplement: S7 Fig — The data were extracted from Schmid et al [50] and displayed using the AtGenExpress Visualization Tool (http://jsp.weigelworld.org/expviz/expviz.jsp). (PDF) [file pgen.1004944.s007.pdf]

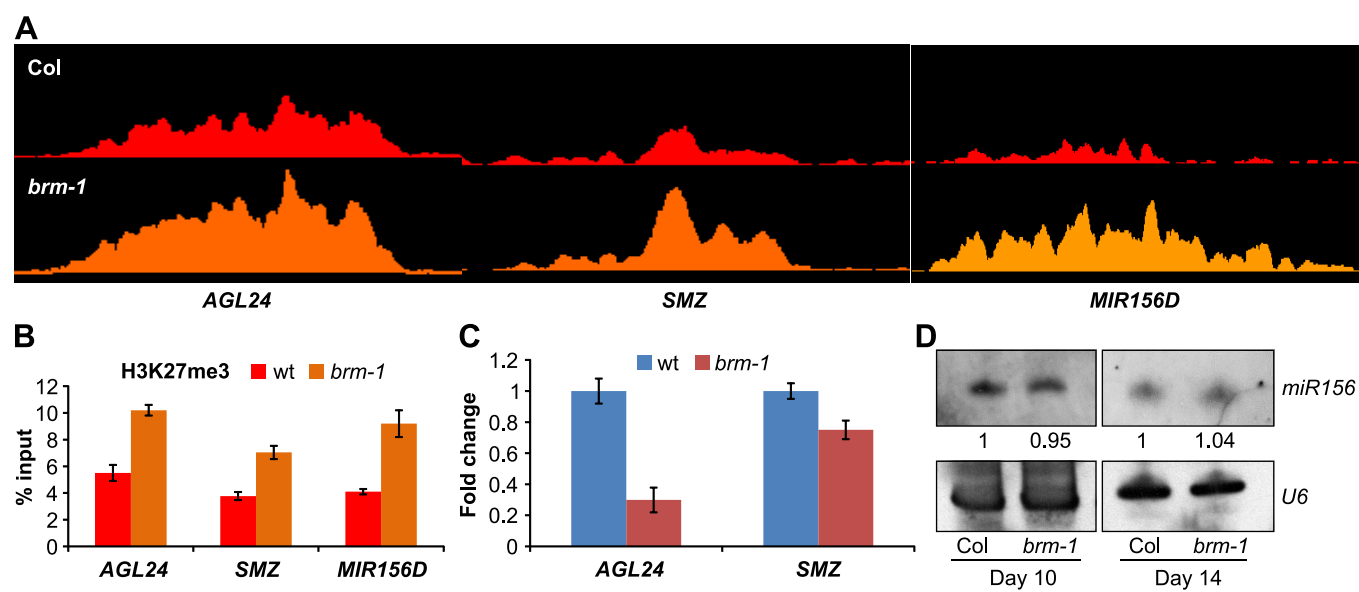

Fig S8

Supplement: S8 Fig — (A) ChIP-seq data showing an increase in H3K27me3 levels at several genes in brm-1. Data from the wild-type Col is shown in red at the top, and brm-1 is shown in orange at the bottom. (B) ChIP-qPCR validation using independent samples. Data are shown as percentage of input. Error bars indicate standard deviations among three technical replicates from one representative experiment. (C) Expression analysis of AGL24 and SMZ by qRT-PCR. The expression of each gene was normalized to that of GAPDH, and the expression level in Col was set to 1. Error bars indicate standard deviations among three technical replicates from one representative experiment. (D) Small RNA northern blot analysis of miR156 in brm-1 compared with Col. Two time points were used (10 days and 14 days after germination). The levels of small RNAs in brm-1 were compared with those in Col, which was set as 1. The numbers below the gel images indicate relative abundance. U6 served as loading control. RNA isolation and hybridization for miRNA detection was performed as described [65]. Digoxigenin-labeled miRNA probes were generated using the mirVana miRNA Probe Construction Kit (Ambion). Oligonucleotide probes used are listed in S2 Table. (PDF) [file pgen.1004944.s008.pdf]
