# Supplementary material for: The Arabidopsis SWI2/SNF2 Chromatin Remodeler BRAHMA Regulates Polycomb Function during Vegetative Development and Directly Activates the Flowering Repressor Gene SVP
Source: PLoS Genet. 2015 Jan 23;11(1):e1004944. doi: 10.1371/journal.pgen.1004944 (PMC4304717; doi:10.1371/journal.pgen.1004944)
Supplement: S1 Table — (PDF) [file pgen.1004944.s009.pdf]

Table S1: Summary of changes in H3K27me3 levels, in CLF occupancy and in gene expression in *brm-1* mutants.

	**H3K27me3 in *brm* mutant**	**CLF binding in *brm* mutant**	**SWN binding in *brm* mutant**	**Expression in *brm* mutant**
AT1G78290	Increase	Increase	Increase	Decrease
AT1G54740	Increase	Increase	Increase	Decrease
AT3G22160	Increase	Increase	Increase	No change
AT4G37540	Increase	Increase	Increase	Decrease
SVP	Increase	Increase	Increase	Decrease
BEL1	Increase	Increase	Increase	Decrease
AT5G33390	Increase	Increase	Increase	No change
TCP2	Increase	Increase	Increase	Decrease
MIR166A	Increase	Increase	Increase	Decrease
WRKY23	Decrease	Decrease	Decrease	Increase
