# Supplementary material for: The Arabidopsis SWI2/SNF2 Chromatin Remodeler BRAHMA Regulates Polycomb Function during Vegetative Development and Directly Activates the Flowering Repressor Gene SVP
Source: PLoS Genet. 2015 Jan 23;11(1):e1004944. doi: 10.1371/journal.pgen.1004944 (PMC4304717; doi:10.1371/journal.pgen.1004944)
Supplement: S2 Table — (PDF) [file pgen.1004944.s010.pdf]

Table S2. Oligonucleotides Used in This Study

Gene-specific primers pairs for qRT-PCR

AT1G06980-F: 5'-AGGACACTCCTCGCCGGAAG-3'
AT1G06980-R: 5'-TCGACGCCGACGACACTACT-3'
AT1G54740-F: 5'-CATGCGGAGGTGGAGACCGA-3'
AT1G54740-R: 5'-TTTCTGCGCTTGCACCACCA-3'
AT3G22160-F: 5'-ACAACCACCACTGCCGGTGA-3'
AT3G22160-R: 5'-CGGACCCGAAAGCCATAGCG-3'
AT4G37540-F: 5'-TCCAACGTCCTGCTTTGTTTCAGT-3'
AT4G37540-R: 5'-TCACAGGAGATCATCAACGACGGA-3'
SVP-F: 5'-TTCCATTTCAGTCGTCTTGTCA-3'
SVP-R: 5'-GAATCTTTTCTCTCGCCATCAC-3'
BEL1-F: 5'-AATCCTACAAGCATCAGTCTCC-3'
BEL1-R: 5'-CTTTCCTTTACTCCAAGACTGC-3'
TCP2-F: 5'-TCTTGACTTCTAAAGGACCACG-3'
TCP2-R: 5'-GGAAAATGAGTGTTGTTGAGTGA-3'
AT5G33390-F: 5'-ATATCCAAATCCCCACAAATA-3'
AT5G33390-R: 5'-AACTAGGTTACGGGTCTGTTC-3'
WRKY23-F: 5'-ACTACCCGTCGTCACAAAGC-3'
WRKY23-R: 5'-CAAAGTCTTGATGCTGCTGAG-3'
BRM-F: 5'-TTTAGAAAGGAAAAGGATTAGGC-3'
BRM-R: 5'-GCCGTTCGCATAACCTCA-3'
AP1-F: 5'-GAAGGCCATACAGGAGCAAA-3'
AP1-R: 5'-ACTGCTCCTGTTGAGCCCTA-3'
FT-F: 5'-CTTGGCAGGCAAACAGTGTATGCAC-3'
FT-R: 5'-GCCACTCTCCCTCTGACAATTGTAGA-3'
FIE-F: 5'-CGTTTCTTCGATGTCTTCGT-3'
FIE-R: 5'-ACGACTCTTCCTTATCTTCATCAG-3'
EMF2-F: 5'-CAGAAGACTGAAGTAACTGAAGAC-3'
EMF2-R: 5'-AAATTGAGGAGATCGTGGGT-3'
VRN2-F: 5'-GCAGAAATAACACCAGGAGAC-3'
VRN2-R: 5'-CCACGGTTTCCATCATTCAG-3'
CLF-F: 5'-ATTATTCGCATGACCCTTGAG-3'
CLF-R: 5'-CATGTCTTGCCTTGATTTCAC-3'
SWN-F: 5'-CAGGGAATGATAATGATGAGGT-3'
SWN-R: 5'-GACCAGCAGACTTTGTAGAG-3'
GAPDH-F: 5'-CTTGGAAGGAGCTAGGAATTGACA-3'
GAPDH-R: 5'-ATGTGTTTCCCTGCACCTTCTC-3'
SMZ-F: 5'-AGGGAGAAGGAGCCATGAAGTTTGGTG-3'

SMZ-R: 5’-GTCTTCAGAGGTTTCATGGTTGCCATG-3’
AGL24-F: 5’-CAGAGTCGGTGACCACAAATG-3’
AGL24-R: 5’-ATGGAAGCCTAACCAACAAAC-3’

Oligonucleotide probes for detecting microRNAs

miR156: 5’-TGACAGAAGAGAGTGAGCACCCTGTCTC-3’
U6: 5’- GAGAAGATTAGCATGGCCCCTCCTGTCTC -3’

Primers for the *XVE::aMIRBRM* transgene construct

BRMmiR-s-I: 5’-GATACAAATTTGCGGTACGGCGTTCTCTCTTTTGTATTCC-3’
BRMmiR-a-II: 5’-GAACGCCGTACCGCAAATTTGTATCAAAGAGAATCAATGA-3’
BRMmiR*s-III: 5’-GAACACCGTACCGCATATTTGTTTCACAGGTCGTGATATG-3’
BRMmiR*a-IV: 5’-GAAACAAATATGCGGTACGGTGTTCTACATATATATTCCT-3’
BRMmiR-A: 5’-GGGGACAAGTTTGTACAAAAAAGCAGGCTTCCTGCAAGGCGATTAAGTTGGGTAAC-3’
BRMmiR-B:
5’-GGGGACCACTTTGTACAAGAAAGCTGGGTCGCGGATAACAATTTCACACAGGAAACAG-3’

Primers used for ChIP-qPCR analyses

AT1G06980-F: 5’- ATGGTCAATCCCGCTTTGGT -3’
AT1G06980-R: 5’- GCCAGACAACACCTTAGGCT -3’
AT1G54740-F: 5’- CATGCGGAGGTGGAGACCGA -3’
AT1G54740-R: 5’- TTTCTGCGCTTGCACCACCA -3’
AT3G22160-F: 5’- GTTTGGGACGGTGGATGGTT -3’
AT3G22160-R: 5’- CGTTTTCTCATTGCAACCTCG -3’
AT4G37540-F: 5’- TTGAGACTGTTCTTCGCGGC -3’
AT4G37540-R: 5’- TCTCCGTCGTGGATCTGGAG -3’
SVP-1-F: 5’-CAACGGCGAGACAAGTGA-3’
SVP-1-R: 5’-GAGAGAAAGAAAGACCTGGAGC-3’
SVP-2-F: 5’- TTCTGAAATGTCATCTTCCCAA -3’
SVP-2-R: 5’- CTGAAGAACATCAAGGGCTACA -3’

SVP-3-F: 5’- CAACGGCGAGACAAGTGA -3’
SVP-3-R: 5’-GAGAGAAAGAAAGACCTGGAGC-3’
SVP-4-F: 5’- GCAGTCAAAGAACTTGGAGAA -3’
SVP-4-R: 5’- GTTACAGAAATCGGGAAAGA -3’
BEL1-F: 5’- TCATGGTATGAGAAGATTTGCCT -3’
BEL1-R: 5’- TCTACAAGAACCCATCTCCAAGA -3’
TCP2-F: 5’-TCTTGACTTCTAAAGGACCACG-3’
TCP2-R: 5’-GGAAAATGAGTGTTGTTGAGTGA-3’
AT5G33390-F: 5’- AATTCCGGCTGCATTTATGGG -3’
AT5G33390-R: 5’- GTACCACAACGTTCCAACAGG -3’
miR156D-F: 5’-TCAAGTCTTTGTTAGTTGCTGTT-3’
miR156D-R: 5’-ATTAGTCCAGAAACCGATGAAA-3’
WRKY23-F: 5’-ACTACCCGTCGTCACAAAGC-3’
WRKY23-R: 5’-CAAAGTCTTGATGCTGCTGAG-3’
ACTIN 2/7-F: 5’-CGTTTCGCTTTCCTTAGTGTTAGCT-3’
ACTIN 2/7-R: 5’-CTTGAAGAAGAAGAAGATGATA-3’
AG-F: 5’-ATGCTGAAGTCGCACTCATCGTCT-3’
AG-R: 5’-GAGCACGAGAAGAAGAAGAAACCTG-3’
TA3-F: 5’-GATTCTTACTGTAAAGAACATGGCATTGAGAGA-3’
TA3-R: 5’-TCCAAATTTCCTGAGGTGCTTGTAACC-3’
AT2G22560-F: TAATGTCCCTAATGTTCCCAAA
AT2G22560-R: CTCAGGCTTACTCAAACCCGA
SMZ-F: 5’-CGAAGATCAAGATCGGAAAGTAC-3’
SMZ-R: 5’-CCGACGAGCATCAGCCAC-3’
AGL24-F: 5’-CTTTGCGATGCTGATGTTG-3’
AGL24-R: 5’-GATCTCCGAGCCTACTGATAAT-3’

Primers used for generating *YFP-SWN* transgene construct
SWN-F:
GGGGACAAGTTTGTACAAAAAAGCAGGCTTCATGGTGACGGACGATAGCAA
CT
SWN- R: GGGGACCACTTTGTACAAGAAAGCTGGGTC
TCAATGAGATTGGTGCTTTCTGGC
